# Pneumolysin Activates the NLRP3 Inflammasome and Promotes Proinflammatory Cytokines Independently of TLR4

**DOI:** 10.1371/journal.ppat.1001191

**Published:** 2010-11-11

**Authors:** Edel A. McNeela, Áine Burke, Daniel R. Neill, Cathy Baxter, Vitor E. Fernandes, Daniela Ferreira, Sarah Smeaton, Rana El-Rachkidy, Rachel M. McLoughlin, Andres Mori, Barry Moran, Katherine A. Fitzgerald, Jurg Tschopp, Virginie Pétrilli, Peter W. Andrew, Aras Kadioglu, Ed C. Lavelle

**Affiliations:** 1 Adjuvant Research Group, School of Biochemistry and Immunology, Trinity College Dublin, Dublin, Ireland; 2 Department of Infection, Immunity and Inflammation, University of Leicester, Leicester, United Kingdom; 3 School of Biochemistry and Immunology, Trinity College Dublin, Dublin, Ireland; 4 Department of Medicine, University of Massachusetts, Worcester, Massachusetts, United States of America; 5 Department of Biochemistry, University of Lausanne, Epalinges, Switzerland; University of Toronto, Canada

## Abstract

Pneumolysin (PLY) is a key *Streptococcus pneumoniae* virulence factor and potential candidate for inclusion in pneumococcal subunit vaccines. Dendritic cells (DC) play a key role in the initiation and instruction of adaptive immunity, but the effects of PLY on DC have not been widely investigated. Endotoxin-free PLY enhanced costimulatory molecule expression on DC but did not induce cytokine secretion. These effects have functional significance as adoptive transfer of DC exposed to PLY and antigen resulted in stronger antigen-specific T cell proliferation than transfer of DC exposed to antigen alone. PLY synergized with TLR agonists to enhance secretion of the proinflammatory cytokines IL-12, IL-23, IL-6, IL-1β, IL-1α and TNF-α by DC and enhanced cytokines including IL-17A and IFN-γ by splenocytes. PLY-induced DC maturation and cytokine secretion by DC and splenocytes was TLR4-independent. Both IL-17A and IFN-γ are required for protective immunity to pneumococcal infection and intranasal infection of mice with PLY-deficient pneumococci induced significantly less IFN-γ and IL-17A in the lungs compared to infection with wild-type bacteria. IL-1β plays a key role in promoting IL-17A and was previously shown to mediate protection against pneumococcal infection. The enhancement of IL-1β secretion by whole live *S. pneumoniae* and by PLY in DC required NLRP3, identifying PLY as a novel NLRP3 inflammasome activator. Furthermore, NLRP3 was required for protective immunity against respiratory infection with *S. pneumoniae*. These results add significantly to our understanding of the interactions between PLY and the immune system.

## Author Summary

*Streptococcus pneumoniae* (pneumococcus) is a pathogen of global significance, causing diseases including pneumonia, meningitis and septicaemia. In order to develop improved pneumococcal vaccines it is essential to understand how the bacterium interacts with the host immune system. Pneumococci produce a range of pathogenicity factors, among which the toxin pneumolysin plays a central role and has potential as a vaccine candidate. Here, we demonstrate that pneumolysin can directly activate innate immune cells and dramatically amplify the production of pro-inflammatory cytokines. These enhancing effects of the toxin do not require Toll-like receptor (TLR)4. In particular, the toxin exerts a potent effect on interleukin (IL)-1, which is an endogenous pyrogen and powerful activator of IL-17A production. This effect results from activation of the NLRP3 inflammasome complex and NLRP3 is required for protection against the pathogen *in vivo*. To induce protective immunity against pneumococci, IFN-γ and IL-17A are thought to be essential. We show that pneumolysin plays a key role in promoting these cytokines both *in vitro* and *in vivo* during respiratory infection. The results add significantly to our understanding of the interactions between pneumococci and the immune system and support investigations into the inclusion of pneumolysin or its derivatives in novel pneumococcal vaccines.

## Introduction

*Streptococcus pneumoniae* is responsible for millions of deaths annually from pneumonia, meningitis and septicaemia while also causing other less serious infections, such as otitis media and sinusitis. Pneumolysin (PLY) is a major virulence factor that is expressed by virtually all clinical isolates of the bacterium. The toxin is a member of the cholesterol-dependent cytolysins, a family that includes perfringolysin O and streptolysin O, expressed by *Clostridium perfringens* and *Streptococcus pyogenes*, respectively. A classical feature of these toxins is their ability to create transmembrane pores in cholesterol-containing membranes and thereby cause cell lysis (reviewed in [1]).

The importance of PLY as a pneumococcal virulence factor is well established, with several studies showing reduced pathogenesis in mice infected with PLY-deficient strains of *S. pneumoniae*, compared to isogenic toxin-producing strains [2], [3], [4], [5]. Furthermore, application of PLY directly into the lungs of rats induced an acute inflammatory response similar to that observed during pneumococcal pneumonia [6]. At sublytic concentrations, the toxin has been reported to promote activation of host complement [7], potentiation of neutrophil activity [8], [9], activation and chemotaxis of CD4^+^ T cells [10] and enhanced production of proinflammatory cytokines in macrophages and monocytes [11], [12]. Studies have been carried out to address the mechanisms underlying the immunomodulatory effects of PLY and particularly the role of TLRs. It has been proposed that the ability of PLY to induce cytokine production [13] and apoptosis [14] in peritoneal macrophages was TLR4-dependent. In contrast, the activation of p38 mitogen-activated protein kinase in epithelial cells [15] and the activation of nuclear factor of activated T cells (NFAT) [16] by PLY were reported to be TLR4-independent. Furthermore, there are conflicting reports on the role of TLR4 in defence against pneumococcal infection. TLR4 was reported to be required for host defence against PLY-producing pneumococci, as mice lacking functional TLR4 were more susceptible to disease after nasopharyngeal challenge [13]. However, other studies have shown a more limited or indeed a redundant role for TLR4 in host resistance to pneumococcal disease, depending on the bacterial dose and the model of infection [17], [18]. Thus, the role of TLR4 in immune responses to the pneumococcus, and particularly to PLY, is unclear and must be resolved.

T cells play a key role in protection against pneumococcal infection [4], [10] so it is important to determine the factors underlying pneumococcus-induced T cell responses. In this regard it is noteworthy that studies in IFN-γ^−/−^ mice indicate a protective role for IFN-γ during bacteremic pneumococcal pneumonia [19] and administration of recombinant IFN-γ to mice promoted protection from disease following intratracheal infection with *S. pneumoniae* [20]. Furthermore, an essential role was proposed for IL-17A in protection against pneumococcal nasopharyngeal colonization following intranasal immunization of mice with killed pneumococci and cholera toxin adjuvant, as protection was abrogated in mice deficient in the IL-17A receptor [21]. Thus, there is a strong rationale for the development of vaccination approaches that induce IFN-γ- and IL-17A-producing cells and for understanding the mechanisms by which pneumococci may either promote or evade such responses.

The activation and differentiation of naïve CD4^+^ T cells following immunization or infection depends on interactions with DC [22]. T cell activation requires antigen presentation on MHC class II molecules, as well as costimulatory signals provided by molecules including CD80 and CD86 on the DC surface. The differentiation of Th1 and Th17 cells requires polarizing cytokines which can be produced by DC. IL-12 and IL-18 are two of the key cytokines involved in Th1 cell differentiation, while IL-23, IL-1 and IL-6 promote Th17 cell development. To date, little is known about the interaction of PLY with DC. Therefore, in this study the effects of PLY on DC maturation and cytokine production and the role of TLR4 in these processes were determined. In addition to the key role of DC in dictating T cell differentiation and polarization, important roles for natural killer (NK) cells and γδ T cells have also been described. In particular, IFN-γ produced by NK cells plays a key role in the instruction of Th1 responses [23], while IL-17A derived from γδ T cells promotes Th17 responses [24].

We demonstrate that endotoxin-free PLY alone does not induce cytokine production by DC or macrophages but it can synergize with TLR agonists to enhance cytokine secretion. Furthermore, PLY promotes the secretion of cytokines including IFN-γ and IL-17A by splenocytes and is essential for *S. pneumoniae* to promote the production of IFN-γ by NK cells and IL-17A by γδ T cells in the lung following respiratory infection. In contrast to previous publications showing a role for TLR4 in PLY-induced immune responses, we show that the ability of the toxin to enhance cytokine secretion does not require TLR4. However, NLRP3 activation was required for PLY- and live *S. pneumoniae*-mediated enhancement of IL-1β secretion and NLRP3 was required for protection against respiratory infection with *S. pneumoniae*.

## Results

### PLY induces TLR4-independent secretion of proinflammatory cytokines by splenocytes

**Figure 1:**
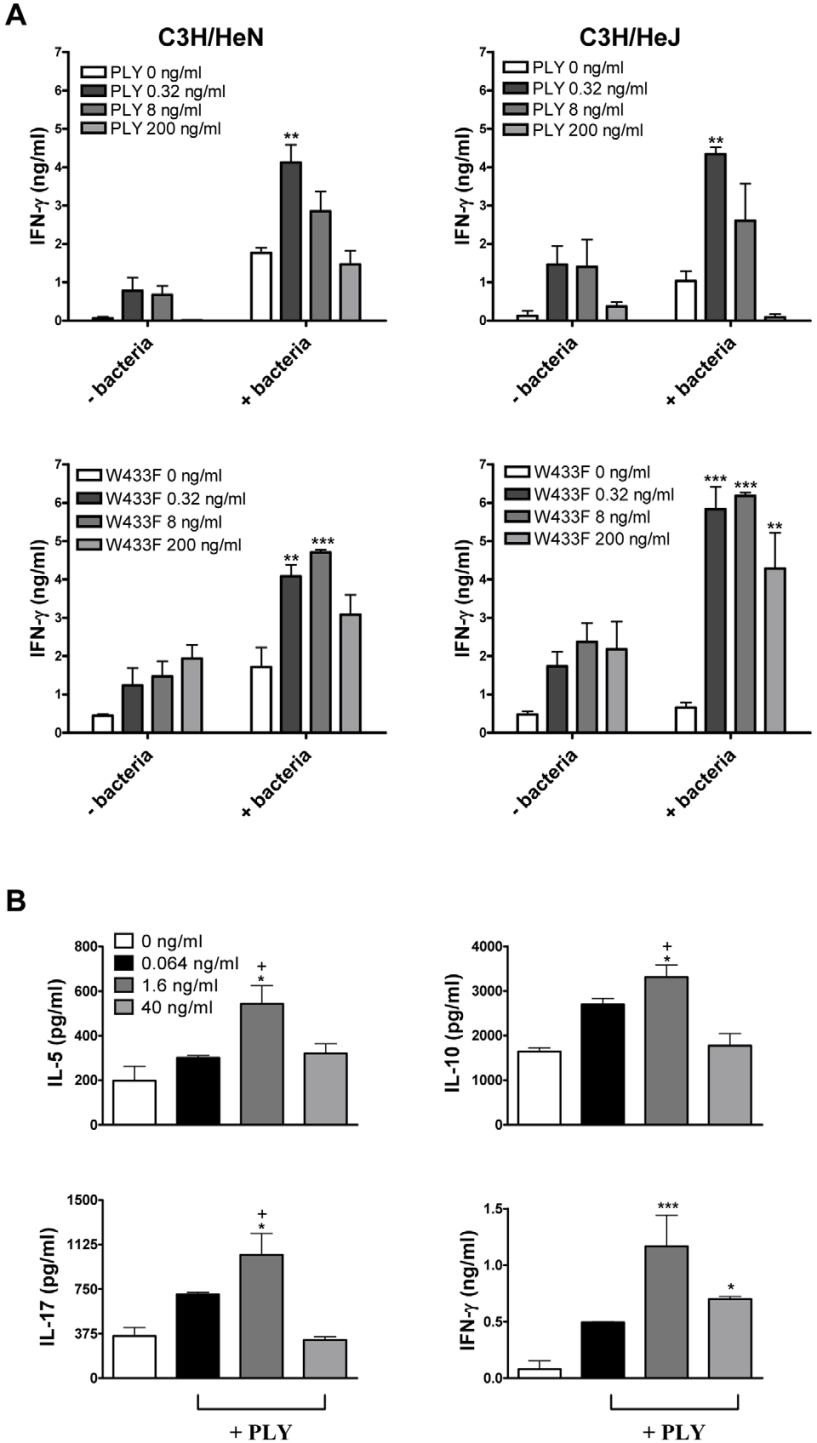
PLY enhances cytokine production by splenocytes independently of TLR4. (A) Spleen cells from C3H/HeN and C3H/HeJ mice were incubated for 72 hours with PLY (0.32–200 ng/ml) (58,500 HU/mg) or W433F in the presence or absence of HkSp (1 bacterium:1 spleen cell). Splenocytes were then stimulated with PMA (5 µg/ml) and ionomycin (300 ng/ml) for a further 24 hours to activate the cells. IFN-γ concentrations were determined in supernatants. ** P<0.01 and *** P<0.001 vs. bacteria + PMA/ionomycin alone. (B) Spleen cells (1×10^6^/ml) from C3H/HeJ mice were incubated with plate-bound anti-CD3 (10 µg/ml) alone or with PLY (0.064–40 ng/ml) (58,500 HU/mg). IFN-γ, IL-5, IL-10 and IL-17A were measured in supernatants after 72 hours. * P<0.05 and *** P<0.001 vs. anti-CD3 alone. +P<0.05 vs. 1.6 ng/ml. All cytokine concentrations are presented as the mean (+ SEM) for triplicate samples.

IFN-γ and IL-17 responses play an essential role in protective immunity against pneumococcal disease [19], [20], [21]. We therefore investigated the ability of PLY to promote these cytokines *in vitro* and the requirement for TLR4. In the absence of any stimulation with PMA or anti-CD3, PLY alone was unable to promote cytokine secretion by splenocytes (data not shown). However, we did find significant enhancing effects of PLY on IFN-γ secretion by splenocytes stimulated with heat-killed *S. pneumoniae* (HkSp) and PMA/ionomycin (Fig. 1A). This was true for spleen cells from both C3H/HeN and C3H/HeJ mice, indicating that the combination of PLY and HkSp is a potent stimulus for TLR4-independent IFN-γ secretion in spleen cells (Fig. 1A). The IFN-γ inducing ability of the toxin was not dependent on its cytolytic activity as a mutant of PLY (W433F) with greatly reduced cytotoxic activity was also capable of stimulating IFN-γ production by splenocytes (Fig. 1A). Indeed, concentrations of W433F from 0.32–200 ng/ml enhanced pneumococci-induced IFN-γ production, whereas the wild-type toxin only significantly augmented IFN-γ secretion at a concentration of 0.32 ng/ml (Fig. 1A). The pore-forming activity of the toxin may, therefore, interfere with its ability to stimulate IFN-γ production in splenocytes.

Furthermore, in the presence of anti-CD3, PLY significantly augmented IFN-γ secretion by splenocytes (Fig. 1B). We also examined the ability of PLY to induce the secretion of other cytokines by anti-CD3-stimulated splenocytes. At a concentration of 1.6 ng/ml, PLY significantly enhanced IL-5 (3-fold; P<0.05), IL-10 (3-fold; P<0.05) and IL-17A (2-fold; P<0.05), in addition to IFN-γ (14-fold; P<0.001), secretion by splenocytes from TLR4 hyporesponsive C3H/HeJ mice (Fig. 1B). These data demonstrate that PLY induces cytokine secretion by spleen cells in a TLR4-independent manner. PLY at 1.6 ng/ml was more effective at promoting cytokine secretion than at 40 ng/ml (Fig. 1B). However, a concentration of 40 ng/ml PLY may induce early apoptosis in splenocytes, as dual staining of these cells with AnnexinV and propidium iodide (PI) revealed slight increases in AnnexinV+/PI- cells following incubation with the toxin for 6 hours (Fig. S1A). These increases were more apparent following stimulation with 200 ng/ml PLY (Fig. S1A). In contrast, when splenocytes were incubated with 200 ng/ml PLY for 24 hours or 96 hours there was no increase in AnnexinV+/PI- cells (data not shown), although an increase in the percentage of PI+ cells was evident (Fig. S1B and S1C). Higher concentrations of PLY (1–5 µg/ml) induced the death of >25% of splenocytes from either C3H/HeN or C3H/HeJ mice after 24 hours (Fig. S1B) and this increased to >50% by 96 hours (Fig. S1C).

### PLY is required for the induction of IFN-γ and IL-17A responses following pneumococcal infection

**Figure 2:**
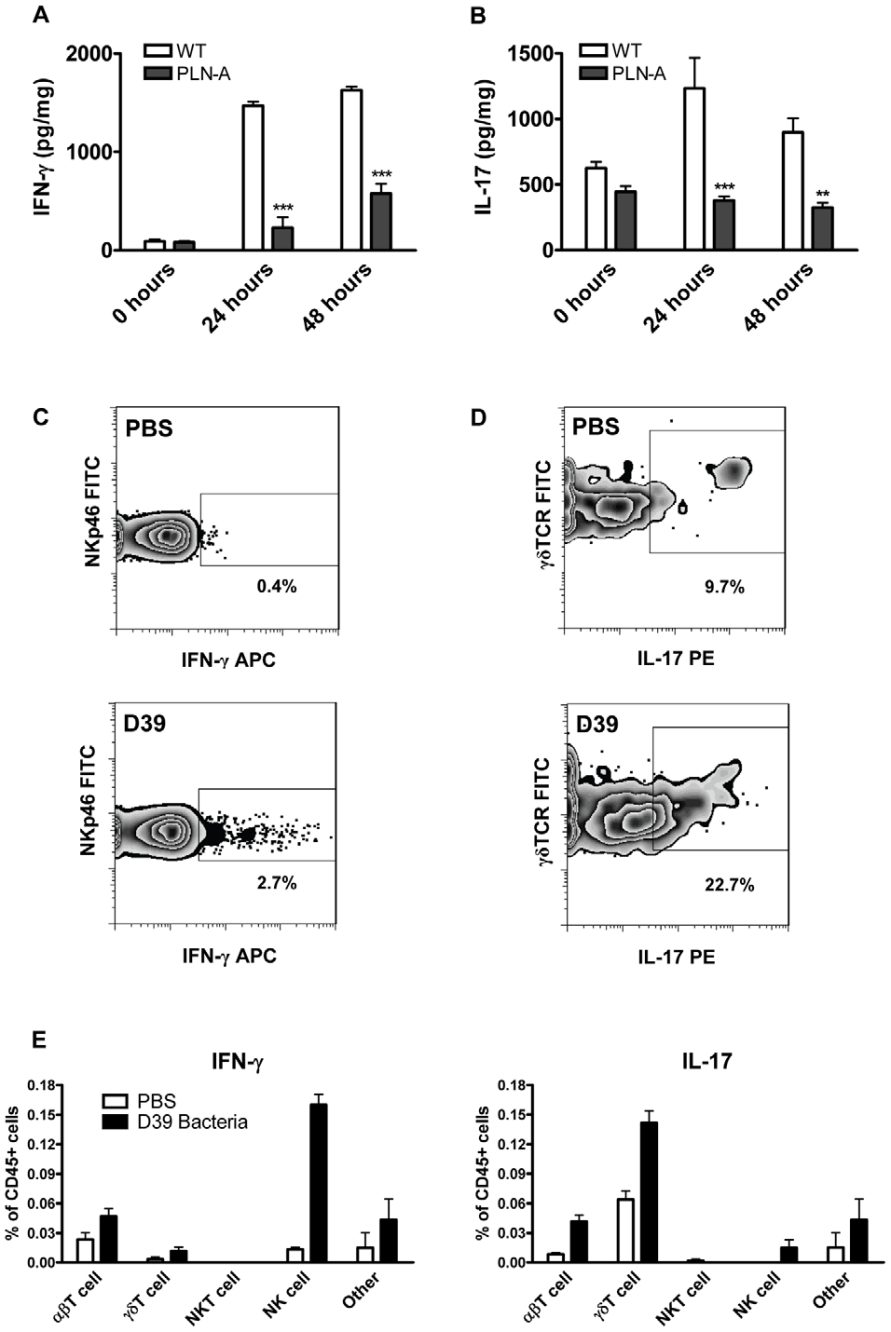
PLY is required for IFN-γ and IL-17A induction following infection with *S. pneumoniae*. (A) IFN-γ concentrations in lungs of infected mice in a model of acute pneumonia. MF1 mice were infected intranasally with wild-type (WT) or PLY-deficient (PLN-A) pneumococci. IFN-γ concentrations were determined in lung homogenates at 0 hours, 24 hours or 48 hours after infection. Results are mean cytokine concentration (+ SEM) for 5 mice per group. *** P<0.001 vs. WT. (B) IL-17A concentrations in lungs of infected mice in a model of resolving pneumonia. BALB/c mice were infected intranasally with WT or PLN-A pneumococci. IL-17A concentrations were determined in lungs at 0 hours, 24 hours or 48 hours after infection. Results are mean cytokine concentration (+ SEM) for 5 mice per group. ** P<0.01 and *** P<0.001 vs. WT. (C–E) Intracellular cytokine staining for IFN-γ and IL-17A in the lungs of *S. pneumoniae* infected mice. Lung cells were isolated from BALB/c mice 48 hours after intranasal infection with wild-type *S. pneumoniae* (D39). Cells were cultured for 5 hours with brefeldin A (GolgiPlug) in the presence of PMA and ionomycin followed by incubation with fluorescently-labelled antibodies against CD3e, CD4, CD8, NKp46, NK1.1, CD45, γδTCR, IFN-γ and IL-17A. Representative dot plots for IFN-γ production by NK cells (panel C; gated on CD4 and NKp46) and IL-17A production by γδ T cells (panel D; gated on side scatter and γδTCR) are shown for control mice (PBS) and pneumococcal-infected mice (D39). Numbers beside gated areas indicate the percentage of positive cells in that area. Histograms show the percentages (n = 6) of various CD45^+^ lung cell populations positive for intracellular IFN-γ or IL-17A (E).

Having shown that PLY enhanced the secretion of cytokines, including IFN-γ and IL-17A by splenocytes stimulated *in vitro* (Fig. 1), we next determined the contribution of PLY to pneumococcus-induced cytokine production using two different murine models of pneumococcal infection *in vivo*. Using a model of acute pneumonia [4], we found that infection with a PLY-deficient strain of pneumococcus (PLN-A) induced significantly less IFN-γ in the lungs of mice compared to infection with its PLY-positive parental strain (Fig. 2A). As has been demonstrated previously in this pneumonia model [3], [4], [5], the PLY-deficient strain exhibited reduced virulence in the lungs compared to the wild-type strain (Fig. S2A). Furthermore, using a model of resolving pneumonia [25], [26], we found significantly lower concentrations of IL-17A in the lungs of mice infected with PLN-A compared to those infected with wild-type pneumococci, at both 24 and 48 hours post-infection (Fig. 2B). There were no significant differences in bacterial CFU at time 0 in the lungs of mice infected with wild-type bacteria compared to PLY-deficient bacteria and lung CFU of both pneumococcal strains were reduced at 24 and 48 hours post-infection (Fig. S2B).

We then investigated the cellular source of these cytokines by intracellular cytokine staining. NK cells were the principal source of IFN-γ in the lungs 48 hours following infection, although IFN-γ was also produced by γδ T cells and other T cells (Fig. 2C and 2E). In the case of IL-17A, the enhanced cytokine production was predominately by γδ T cells with limited IL-17A production in other T cells and NK cells (Fig. 2D and 2E).

### PLY promotes dendritic cell maturation but not cytokine production

Having shown that PLY promotes IFN-γ and IL-17A production *in vitro* and *in vivo*, we next established the effects of the toxin on DC. DC play an important role in the activation and polarization of naïve T cells but to date the effects of PLY on DC have not been widely investigated. We therefore examined the effects of PLY on DC cytokine production and costimulatory molecule expression.

**Figure 3:**
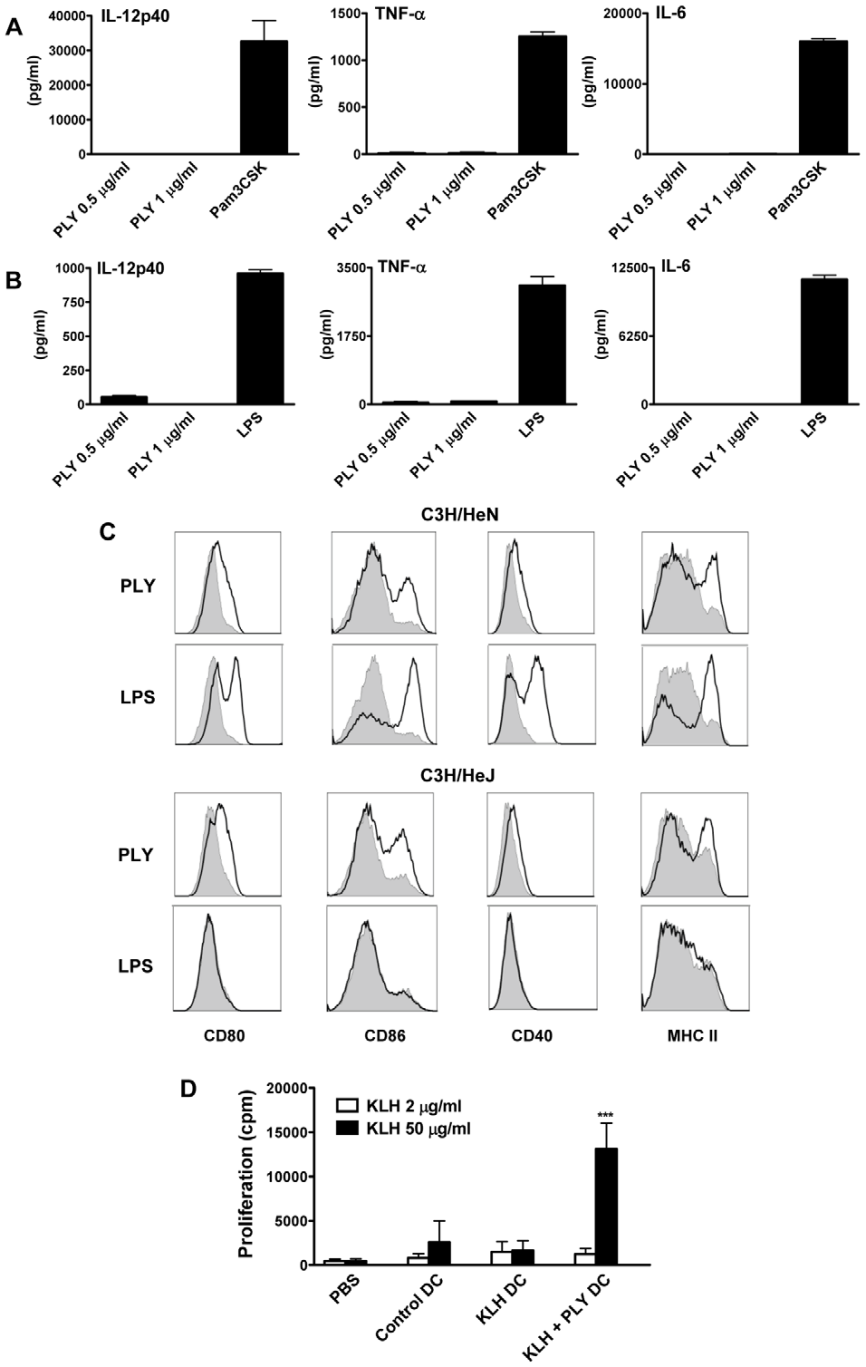
Endotoxin-free PLY does not induce cytokine production by DC or macrophages but does enhance DC maturation in a TLR4-independent manner. DC (A) or BMDM (B) from C57BL/6 mice were incubated with PLY (1 µg/ml or 0.5 µg/ml) or Pam3CSK (10 µg/ml) or LPS (500 pg/ml) for 24 hours. Supernatants were analyzed for IL-12p40, TNF-α and IL-6. Results are presented as mean cytokine concentrations (+ SEM) for triplicate samples. (C) DC from C3H/HeN or C3H/HeJ mice were incubated with medium, PLY (1 µg/ml) or LPS (500 pg/ml). After 24 hours, cells were washed and stained with antibodies specific for CD80, CD86, CD40 and MHC Class II. Immunofluorescence is shown for PLY- or LPS-treated DC (black line) compared to untreated cells incubated with medium (grey histograms). Plots are representative of three independent experiments. (D) Adoptive transfer of DC incubated with antigen and PLY promotes antigen-specific T cell responses. BALB/c mice were immunized subcutaneously in the footpad with DC (5×10^5^ cells/mouse) that had been incubated overnight with medium only as a control or with KLH antigen (10 µg/ml) in the presence or absence of PLY (1 µg/ml). 7 days later splenocytes were isolated and stimulated *ex vivo* with KLH (2 or 50 µg/ml). Proliferation was measured by [^3^H]-thymidine incorporation after 4 days of culture and is expressed as mean cpm (+ SEM; n = 5). *** P<0.001 vs. adoptive transfer of DC stimulated with KLH alone.

While the TLR2 agonist PAM3Csk4 promoted the secretion of proinflammatory cytokines from DC, endotoxin-free PLY, at concentrations of up to 1 µg/ml, did not induce the secretion of IL-6, IL-12p40 or TNF-α (Fig. 3A). Higher concentrations were not tested because PLY at 2 µg/ml caused some cell death in DC, as determined by PI staining (Fig. S3A). Indeed, concentrations of PLY ranging from 3 µg/ml to 6 µg/ml were highly toxic to DC, with PLY at 6 µg/ml inducing death in approximately 90% of DC from either C3H/HeN or C3H/HeJ mice after 24 hours (Fig. S3A and S3B). AnnexinV/PI staining of DC incubated with this higher concentration of the toxin for 6 hours indicated that PLY induced limited apoptosis (AnnexinV+/PI−), but greatly increased the percentage of dead AnnexinV+/PI+ cells (Fig. S3C).

Since previous studies demonstrated that PLY could directly induce cytokine production in monocytes and macrophages [12], [13], it was important to establish if the finding that endotoxin-free PLY does not promote cytokine secretion was specific to DC. Endotoxin-free PLY was also unable to induce secretion of significant concentrations of IL-6, TNF-α, IL-12p40 (Fig. 3B), IL-1β or IL-23 (data not shown) from murine bone-marrow derived macrophages (BMDM). These data indicate that PLY alone does not stimulate inflammatory cytokine production by DC or macrophages.

Since co-stimulation is required for DC to promote T cell activation, we assessed the effects of PLY on the expression of co-stimulatory molecules and MHC class II on DC and compared these effects to those induced by the TLR4 agonist LPS. Stimulation of DC with endotoxin-free PLY enhanced the expression of MHC class II and co-stimulatory molecules, particularly CD86. Interestingly, similar findings were observed with PLY-stimulated DC from both C3H/HeN and LPS-hyporesponsive C3H/HeJ mice, indicating that the enhancement of DC maturation by the toxin was TLR4-independent. In contrast, LPS strongly enhanced the expression of co-stimulatory molecules on DC from C3H/HeN mice only (Fig. 3C). Therefore, although endotoxin-free PLY does not induce cytokine secretion by DC, it can promote their maturation independently of TLR4.

To determine the functional significance of the direct effects of PLY on DC, cells were incubated with antigen (KLH) alone or KLH and PLY overnight and injected into mice. The exposure of DC to PLY increased their ability to promote antigen-specific T cell proliferation in splenocytes 7 days following DC transfer (Fig. 3D). We did not detect enhanced antigen-specific cytokine production by these cells (data not presented). However, the T cell proliferation data indicate that PLY can act directly on DC to enhance their T cell stimulatory activity.

Since exposure of DC to PLY enhanced their ability to promote adaptive immune responses, we determined the adjuvant properties of endotoxin-free PLY when co-injected with KLH. Injection of PLY with KLH did not enhance antigen-specific cellular immune responses (Fig. S4A). In contrast, antigen-specific antibody responses were significantly enhanced by co-immunization with antigen and PLY (Fig. S4B and S4C). The ability of PLY to promote antigen-specific antibody responses applied to both IgG1 and IgG2a subclasses and the toxin was equally effective in C3H/HeN and C3H/HeJ mice. Thus, the toxin has the ability to enhance antigen-specific immune responses independently of TLR4 signalling.

### PLY synergizes with TLR agonists to enhance secretion of pro-inflammatory cytokines

**Figure 4:**
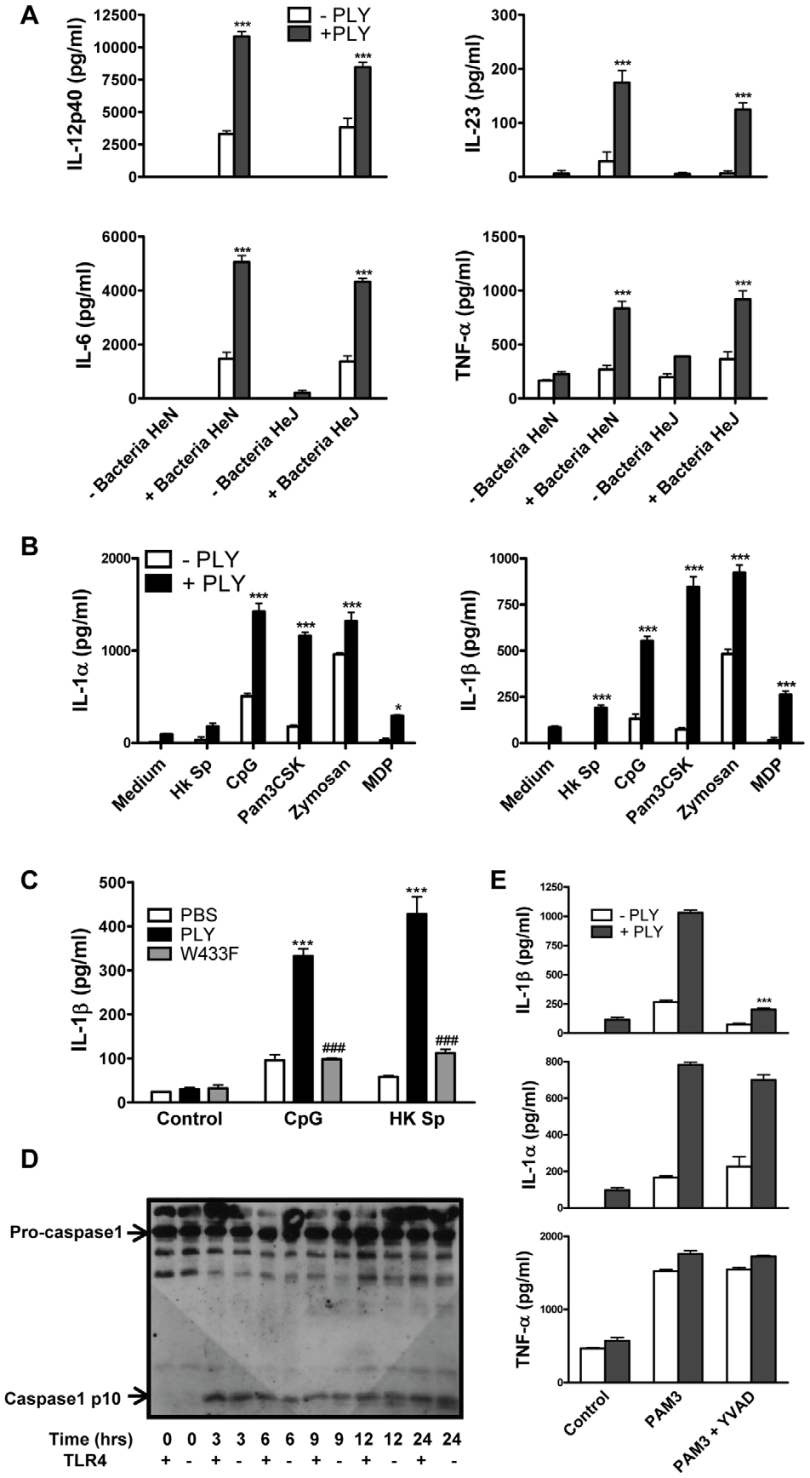
PLY synergizes with TLR agonists to enhance pro-inflammatory cytokine secretion by DC. (A) DC from C3H/HeN or C3H/HeJ mice were incubated with PLY (1 µg/ml) for 1 hour before the addition of HkSp (10 bacteria:1 DC). After 24 hours supernatants were assayed for IL-12p40, IL-6, IL-23 and TNF-α. Results are mean cytokine concentrations (+ SEM) for triplicate samples. *** P<0.001 vs. bacteria alone. (B) DC from C3H/HeJ mice were incubated with PLY (1 µg/ml) for 1 hour before stimulation with TLR or NLR agonists; HkSp (10 bacteria:1 DC), CpG (4 µg/ml), Pam3CSK (10 µg/ml), zymosan (20 µg/ml) or MDP (20 µg/ml). Supernatants were assayed for IL-1α and IL-1β after 24 hours. Results are mean cytokine concentrations (+ SEM) for triplicate samples. * P<0.05 and *** P<0.001 vs. agonist alone. (C) Haemolytic activity is required for the promotion of IL-1β secretion by PLY. DC from C57BL/6 mice were incubated with medium alone (control), or with PLY or W433F for 1 hour before stimulation with CpG (4 µg/ml) or HkSp (10 bacteria:1 DC). Following 24 hours incubation, supernatants were assayed for IL-1β. Results are mean cytokine concentrations (+ SEM) for triplicate samples. ***, P<0.001 vs. agonist alone, ### P<0.001 vs. PLY + agonist. (D) Activation of caspase-1 by PLY. DC (1×10^6^/ml) from C3H/HeN (represented as TLR4 +) or C3H/HeJ (represented as TLR4 −) mice were stimulated with PLY (2 µg/ml) (73,143 HU/mg) for 3, 6, 9, 12 or 24 hours or with medium (0 hours) as a control. Cell lysates were analysed for caspase-1 p10 expression by Western blot. (E) DC from C3H/HeJ mice were incubated with or without the caspase-1 inhibitor YVAD-fmk (40 µM) for 30 minutes before the addition of PLY (1 µg/ml) either alone or 1 hour before Pam3CSK (10 µg/ml). After 24 h supernatants were assayed for IL-1β, IL-1α and TNF-α by ELISA. *** P<0.001 vs. PAM3 + PLY alone.

Although PLY alone did not induce cytokine production by DC or macrophages, we investigated if the toxin could synergize with other stimuli to enhance cytokine secretion. We found that PLY (1 µg/ml) synergized with HkSp to significantly enhance the secretion of IL-6, IL-12p40, IL-23 and TNF-α by DC (Fig. 4A). Notably, this synergistic effect was observed in DC from both C3H/HeN and C3H/HeJ mice, indicating that the enhancement of cytokine secretion by PLY did not require the presence of functional TLR4. Furthermore, PLY was also able to synergize with HkSp to significantly enhance IL-6 and TNF-α production in BMDM (Fig. S5). PLY alone did not induce significant secretion of IL-1α or IL-1β by DC (Fig. 4B) or BMDM (data not shown). However, PLY synergized with HkSp to significantly enhance IL-1β secretion by DC (Fig. 4B). Importantly, the ability of PLY to promote IL-1β secretion was not limited to pneumococci as the toxin also significantly enhanced IL-1β secretion in response to a range of TLR and NLR agonists; PAM3Csk (TLR1/2 ligand), zymosan (TLR2/6 ligand), CpG (TLR9 ligand) and MDP (Nod2 ligand). Similarly, IL-1α secretion was significantly enhanced in DC stimulated with these TLR/NLR agonists and PLY compared to the agonists alone. Furthermore, by using DC from C3H/HeJ mice, we showed that PLY-induced enhancement of IL-1α and IL-1β secretion by TLR agonists was independent of TLR4 (Fig. 4B).

### PLY activates the NLRP3 inflammasome

Since it has been shown that IL-1β is required for resistance to pneumococcal infection [27], we investigated the mechanism underlying PLY-induced IL-1β further. Firstly, to determine the importance of haemolytic activity in the promotion of IL-1β by PLY, DC were incubated with HkSp or CpG in the presence or absence of PLY or the W433F hemolysin mutant. PLY-driven IL-1β secretion was hemolysin-dependent as the ability of W433F to promote secretion of the cytokine was significantly less than wild-type PLY (Fig. 4C).

**Figure 5:**
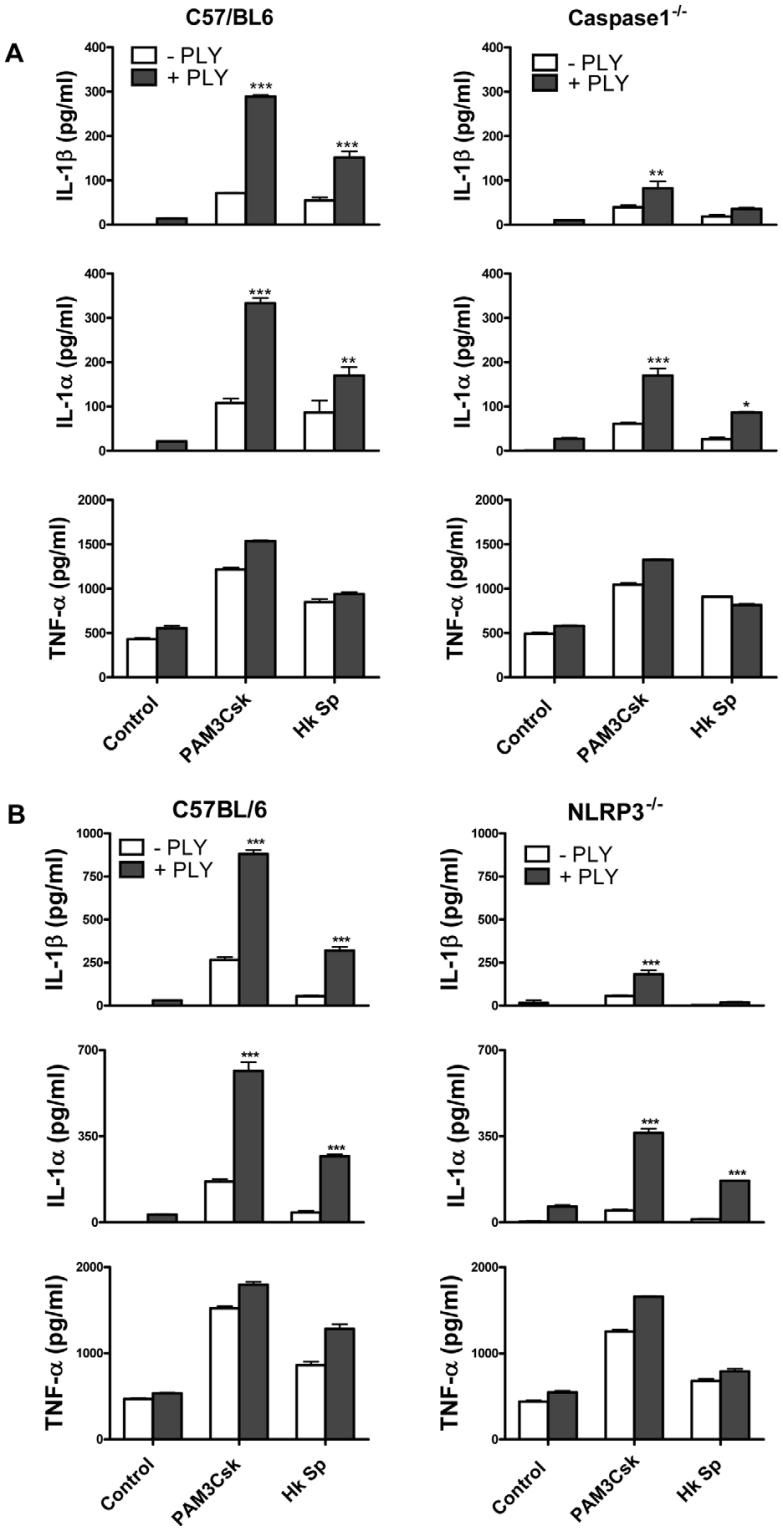
The ability of PLY to enhance IL-1β secretion by DC is caspase-1 and NLRP3-dependent. (A) DC from wild-type C57BL/6 or Caspase-1^−/−^ were incubated with PLY (0.5 µg/ml) for 1 hour before the addition of Pam3CSK (10 µg/ml) or HkSp (10 bacteria:1 DC). IL-1β, IL-1α and TNF-α concentrations were quantified in supernatants after 24 hours and are presented as mean values (+ SEM) from triplicate cultures. * P<0.05, ** P<0.01 and *** P<0.001 vs. agonist alone. (B) DC from wild-type C57BL/6 or NLRP3^−/−^ mice were incubated with PLY (0.5 µg/ml) for 1 hour before the addition of Pam3CSK (10 µg/ml) or HkSp (10 bacteria:1 DC). After 24 h supernatants were assayed for IL-1β, IL-1α and TNF-α by ELISA. *** P<0.001 vs. agonist alone.

IL-1β is synthesised inside cells as a biologically inactive precursor that is processed by caspase-1 prior to its secretion. Caspase-1 is itself produced as an inactive zymogen that must be cleaved to generate the active p10 and p20 subunits. Since the secretion of IL-1β requires both the transcription of IL-1β and caspase-1 activation, we investigated how PLY promoted IL-1β secretion in DC. We found that PLY alone promoted caspase-1 activation in DC and that this was independent of TLR4, as processed (p10) caspase-1 was detected in DC lysates from both C3H/HeN and C3H/HeJ mice (Fig. 4D). Pre-treatment of DC with the caspase-1 inhibitor YVAD-fmk completely blocked the synergy between PLY and PAM3Csk for IL-1β secretion (Fig. 4E), confirming the importance of caspase-1 in PLY-induced IL-1β secretion. In contrast, the enhancement of IL-1α secretion by the toxin was not significantly affected, indicating that caspase-1 was not required (Fig. 4E). The importance of caspase-1 in PLY-induced IL-1β secretion was also confirmed using DC from caspase-1 knockout mice, as the synergy between PLY and either PAM3Csk or HkSp for IL-1β was dramatically reduced in caspase-1^−/−^ DC compared to wild-type DC (Fig. 5A).

**Figure 6:**
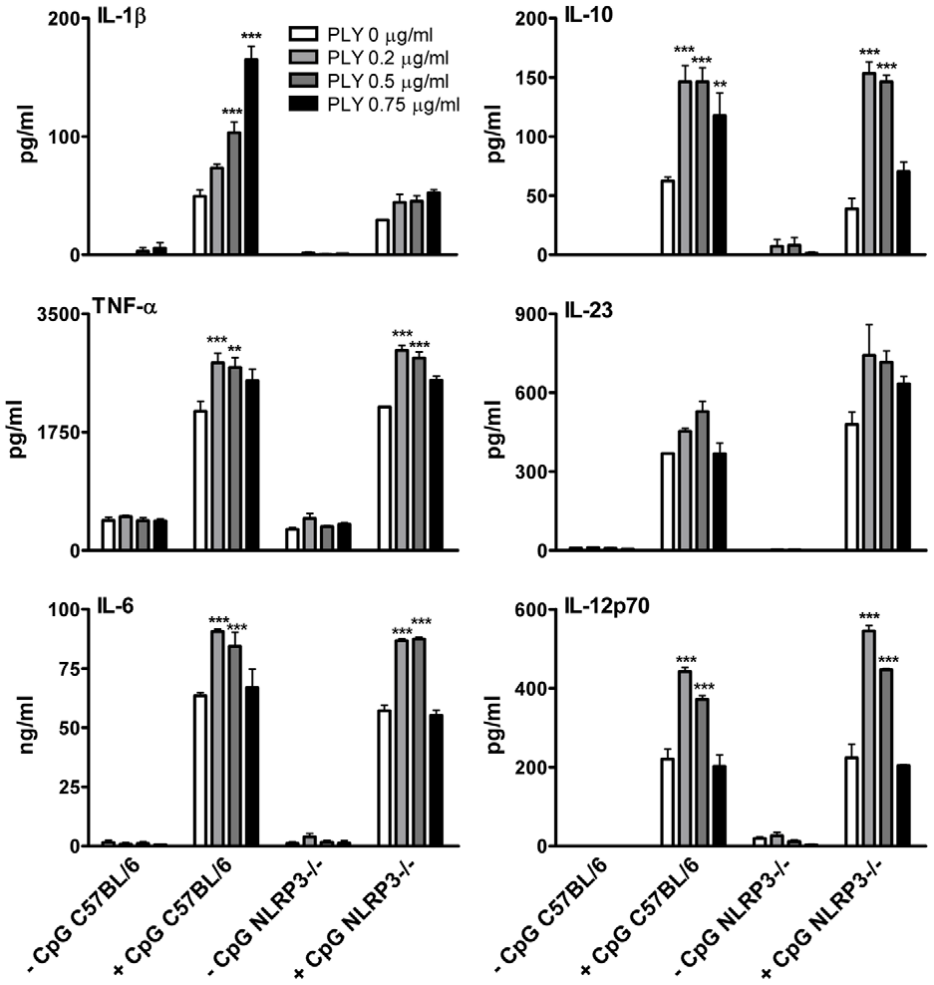
In contrast to PLY-induced IL-1β secretion, the enhancement of TNF-α, IL-6, IL-10 and IL-12 by PLY is independent of NLRP3. DC (6.25×10^5^/ml) from C57BL/6 or NLRP3^−/−^ mice were incubated with medium alone or with various concentrations of PLY (0.2–0.75 µg/ml) for 1 hour before the addition of CpG (4 µg/ml). After 24 hours supernatants were assayed for IL-1β, TNF-α, IL-6, IL-23, IL-10 and IL-12p70. Results are mean cytokine concentrations (+ SEM) for triplicate samples. ** P<0.01 and *** P<0.001 vs. CpG alone.

The activation of caspase-1 is regulated by the assembly of inflammasomes, cytoplasmic multiprotein complexes that contain a nucleotide-binding oligomerization domain (NOD)-like receptor (NLR) family member, such as NLRP3 or NLRC4 and caspase-1 [28]. We investigated the role of the NLRP3 inflammasome in PLY-driven IL-1β secretion and found that the ability of PLY to promote IL-1β secretion was compromised in cells from NLRP3^−/−^ mice compared to wild-type mice (Fig. 5B), but not in DC from mice deficient in NLRP6 or NLRP12 (Fig. S6). In particular, the synergy between PLY and HkSp for IL-1β secretion was abrogated in DC from NLRP3^−/−^ mice (Fig. 5B). This marked defect in the ability of NLRP3^−/−^ DC to secrete IL-1β in response to stimulation with PLY was due to differences in IL-1β processing, as Western blot analysis did not reveal altered proIL-1β production in DC from NLRP3^−/−^ mice compared to DC from wild-type mice (Fig. S7). In contrast to IL-1β secretion, PLY significantly enhanced bacteria-induced IL-1α secretion in DC from both wild-type and NLRP3^−/−^ mice (Fig. 5B). The adapter protein ASC (apoptosis-associated speck like protein containing CARD) is necessary for caspase-1 activation via NLRP3. PLY-mediated enhancement of IL-1β secretion was also reduced in DC from ASC^−/−^ mice, compared to wild-type mice (Fig. S8). DC from caspase-1^−/−^ (Fig. 5A) or NLRP3^−/−^ (Fig. 5B) mice secreted comparable concentrations of TNF-α to wild-type mice when stimulated with PLY and TLR agonist, indicating that the ability of these cells to produce inflammatory cytokines was not globally compromised. Indeed, the ability of PLY to promote the secretion of TNF-α, IL-6, IL-23, IL-12 and IL-10 was independent of NLRP3 (Fig. 6). CpG was used as the TLR for these experiments as it was the most effective TLR agonist to determine synergy with PLY for this panel of cytokines, particularly IL-12p70. The IL-10 data indicate that PLY can promote secretion of both pro- and anti-inflammatory cytokines, although the overall profile suggests that PLY is a highly pro-inflammatory factor.

**Figure 7:**
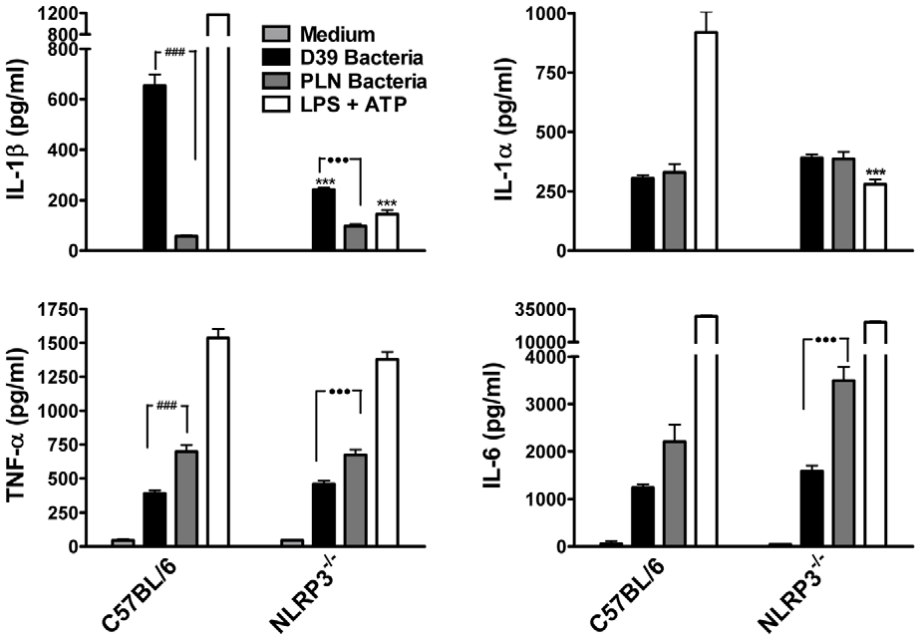
Live *S. pneumoniae* promotes IL-1β secretion by DC in a NLRP3-dependent manner and this requires PLY. DC (6.25×10^5^/ml) from C57BL/6 or NLRP3^−/−^ mice were incubated for 24 hours with medium alone, with wild-type *S. pneumoniae* (D39; 10 bacteria:1DC) or with PLY-deficient bacteria (PLN; 10 bacteria:1 DC). As a positive control for IL-1β secretion and NLRP3 inflammasome activation, DC were primed with LPS (100 ng/ml) for 23 hours prior to one hour stimulation with ATP (2.7 mg/ml). Following incubation, supernatants were removed and assayed for IL-1β, IL-1α, TNF-α and IL-6. Results are mean cytokine concentrations (+ SEM) for triplicate samples. *** P<0.001, NLRP3^−/−^ vs. C57BL/6 DC stimulated with the same treatments, ### P<0.001, D39 vs. PLN bacteria in C57BL/6 DC, ••• P<0.001, D39 vs. PLN bacteria in NLRP3^−/−^ DC.

Having demonstrated that purified endotoxin-free PLY can promote IL-1β secretion via the NLRP3 inflammasome, we next investigated the role of NLRP3 in IL-1β production by DC in response to live *S. pneumoniae*. Wild-type *S. pneumoniae* induced robust IL-1β secretion by DC and this was dependent on PLY since the PLY-deficient strain induced very little IL-1β secretion (Fig. 7). In contrast, IL-1α, TNF-α and IL-6 secretion in response to the PLY-deficient strain was comparable to, or greater than, that induced by the wild-type bacteria. Thus, PLY appears to play a vital and selective role in pneumococcus-induced IL-1β secretion by DC. Furthermore, the induction of IL-1β secretion by the pneumococcus was strongly dependent on NLRP3 (Fig. 7).

**Figure 8:**
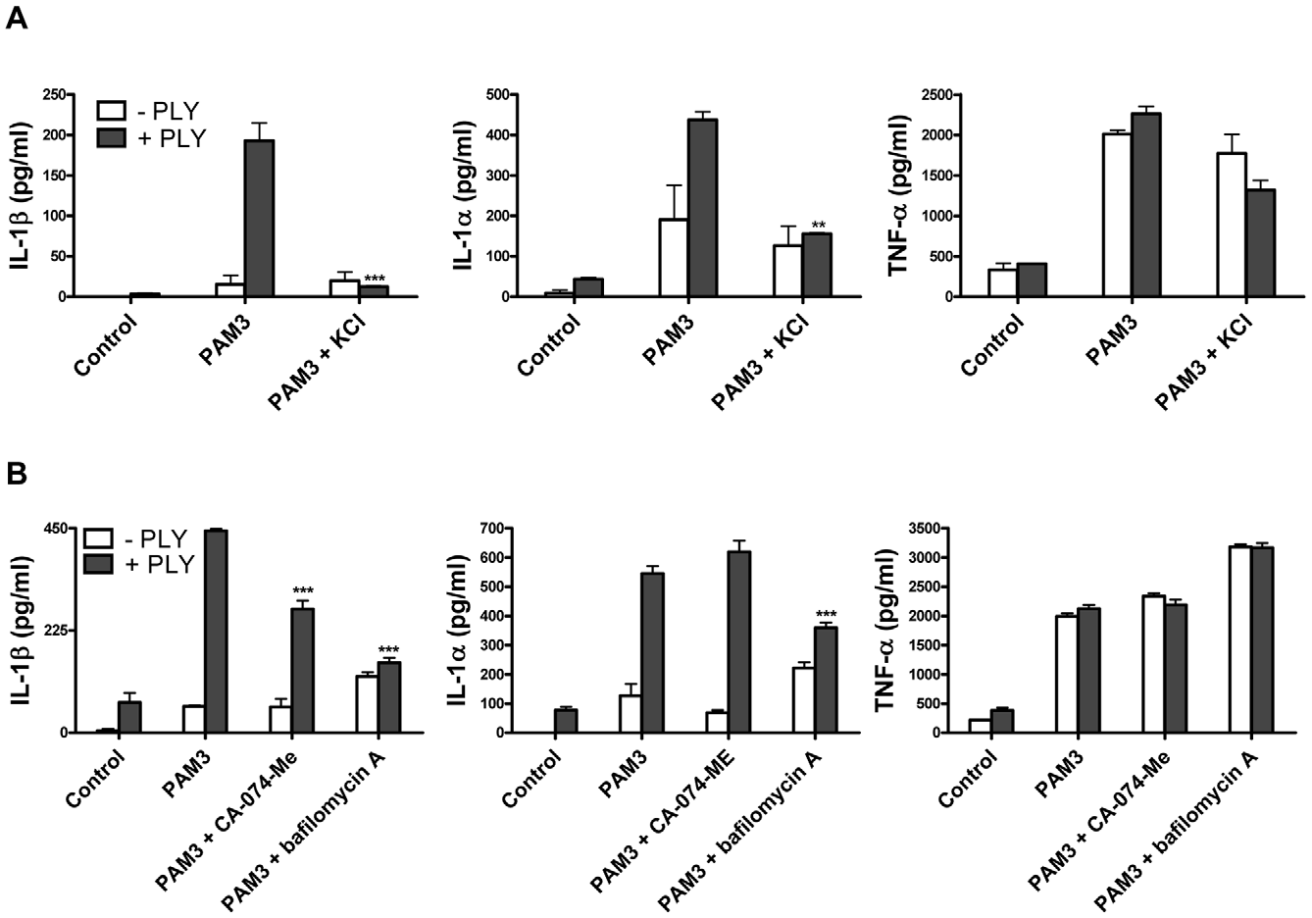
PLY-induced IL-1β secretion by DC is dependent on potassium efflux and phagosomal rupture. (A) DC from C3H/HeJ mice were incubated with medium alone or supplemented with KCl (50 mM) for 30 minutes before addition of PLY (1 µg/ml) either alone or 1 hour before Pam3CSK (10 µg/ml). After 24 hours, supernatants were assayed for IL-1β, IL-1α and TNF-α. ** P<0.01 and *** P<0.001 vs. PAM3 + PLY. (B) DC from C3H/HeJ mice were incubated with a cathepsin B inhibitor CA-074-Me (10 µM) or bafilomycin A (250 nM) for 30 minutes before addition of PLY (1 µg/ml) either alone or 1 hour before PAM3Csk (10 µg/ml). After 24 hours, supernatants were assayed for IL-1β, IL-1α and TNF-α. *** P<0.001 vs. PAM3 + PLY. All cytokine concentrations are presented as the mean (+ SEM) for triplicate samples.

In order to further investigate the mechanisms by which PLY may trigger caspase-1 activation and IL-1β secretion in DC, we examined the involvement of some known factors implicated in NLRP3 inflammasome activation such as reduced cytoplasmic potassium concentration. It has been reported that the pore forming toxins, *Staphylococcus aureus* α-toxin [29] and *Aeromonas hydrophilia* aerolysin [30] induce the secretion of IL-1β and the activation of caspase-1, respectively, by allowing the efflux of intracellular potassium (K^+^). We therefore assessed whether K^+^ efflux could also be the trigger for caspase-1 activation by PLY. The addition of extracellular KCl to the medium (50 mM) significantly inhibited IL-1β secretion by DC in response to PLY and PAM3Csk (Fig. 8A). Interestingly, IL-1α secretion was also significantly reduced in the presence of high potassium concentrations (Fig. 8A).

It has been proposed that the NLRP3 inflammasome may be activated by lysosomal damage and the subsequent release of cathepsin B into the cytoplasm of cells [31]. We investigated the effect of inhibiting cathepsin B on PLY-induced IL-1 secretion and found that inhibition with the specific cathepsin B inhibitor CA-074-Me resulted in a significant reduction in IL-1β, but not IL-1α, secretion by DC stimulated with PLY and PAM3CSk (Fig. 8B). Furthermore, treating DC with bafilomycin A, which inhibits the H^+^ ATPase system required for lysosomal acidification [32], significantly reduced the enhancement of IL-1β and IL-1α, but not TNF-α, by PLY (Fig. 8B).

### The NLRP3 inflammasome is required for protection against *S. pneumoniae* infection

**Figure 9:**
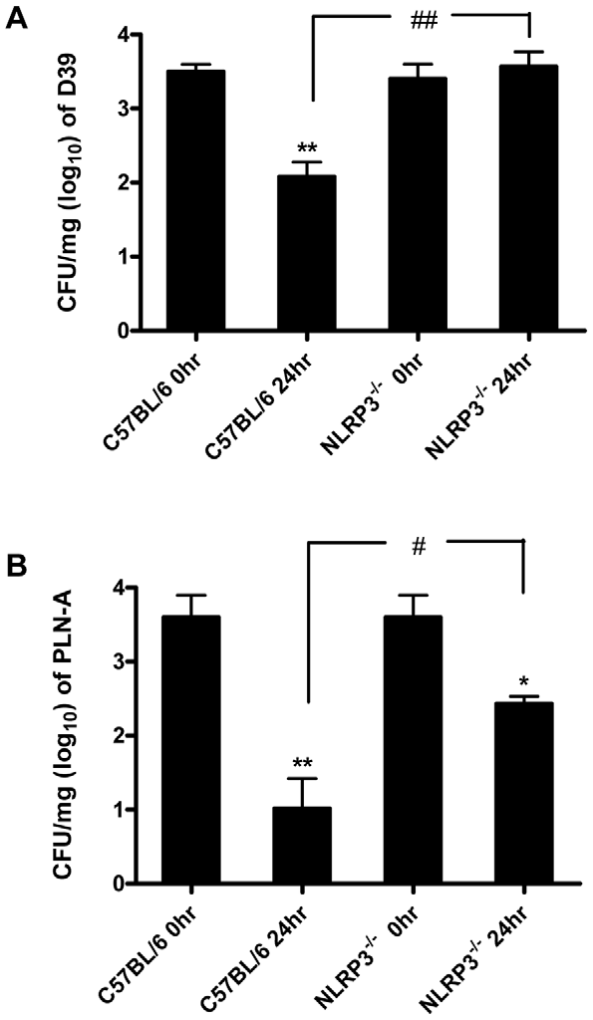
The NLRP3 inflammasome is required for protection against *S. pneumoniae* infection. C57BL/6 and NLRP3^−/−^ mice were nasally infected with 1×10^6^ CFU of wild-type *S. pneumoniae* (strain D39) or PLY-deficient *S. pneumoniae* (PLN-A). Mice were sacrificed at 0 and 24 hours post-infection and bacterial CFU in lung homogenates were assessed. Data are expressed as mean (+ SEM) CFU per mg of lung tissue (log_10_) for five mice per group. * P<0.05 and ** P<0.01 vs. 0 hours p.i. in the same mouse strain, # P<0.05 and ## P<0.01 C57BL/6 24 hours p.i. vs. NLRP3^−/−^ 24 hours p.i. (A) Infection with wild-type bacteria. (B) Infection with PLY-deficient bacteria.

To investigate the significance of PLY and pneumococcal-induced inflammasome activation, NLRP3^−/−^ and wild-type control C57BL/6 mice were intranasally infected with *S. pneumoniae* (strain D39). In contrast to control mice, which showed a significant reduction in lung CFU 24 hr post-infection, bacterial clearance was significantly compromised in NLRP3^−/−^ mice and no reduction in bacterial CFU was evident in the lungs of these mice at this time-point (Fig. 9A).

To determine the role of PLY, mice were also infected with a PLY-deficient isogenic mutant strain of D39 (PLN-A). Bacterial CFU of PLN-A in lungs of control C57BL/6 mice at 24 hr post-infection were significantly lower than in lungs of NLRP3^−/−^ mice (Fig. 9B). In addition, although there were reductions in bacterial CFU of PLN-A in lungs of both NLRP3^−/−^ and control mice over time, the reduction in control mice was significantly greater than observed in NLRP3^−/−^ mice (Fig. 9B). Therefore, NLRP3 appears to play a particularly important role in infection with PLY-expressing *S. pneumoniae*, although the data with PLN-A suggest that even in the absence of PLY, NLRP3 may play a role in protective immunity.

These data indicate for the first time a role for NLRP3 in protection against *S. pneumoniae* infection and this is the first description of a role for NLRP3 in protection against a Gram-positive bacterial pathogen.

## Discussion

In mice, the cytokines IL-1β [27], IFN-γ and IL-17A [19], [20], [21] have been shown to play important protective roles in immunity against pneumococci. Here we show that PLY strongly enhances the secretion of IFN-γ by splenocytes *in vitro* and is required for IFN-γ and IL-17A responses during pneumococcal infection *in vivo*. NK cells are the principal cellular source of IFN-γ in the lungs following infection while γδ T cells are the major producers of IL-17A. This supports recent findings that γδ T cells are a key source of IL-17A production *in vitro* and *in vivo* in response to IL-1 stimulation [24] and pathogen-derived products [33]. The latter study found that IL-17-producing γδ T cells share a number of characteristic features with Th17 cells. IL-1 and IL-23 play key roles in the induction of IL-17A production by γδ T cells and in the differentiation and expansion of Th17 cells [24]. Other cytokines, including IL-6 and TNF-α, have also been shown to be important in Th17 cell differentiation [34], [35], [36]. Importantly, our data show that PLY can synergize with TLR agonists to enhance the secretion of IL-1α, IL-1β, IL-23, TNF-α and IL-6 by DC, which can induce IL-17A production by γδ T cells and promote the differentiation and expansion of Th17 cells. The ability of Freund's complete adjuvant or LPS to induce antigen-specific Th17 cell responses *in vivo* requires IL-1R1, indicating a key role for IL-1 in adjuvant-driven Th17 cell responses [36].

Pneumolysin also strongly enhanced TLR agonist-induced IL-12 secretion, which together with cytokines such as IFN-γ and IL-18, is important for Th1 differentiation and stabilization [37]. Like IL-1β, active IL-18 also requires cleavage of its precursor form by caspase-1 and enhanced levels have been reported in macrophages co-stimulated with recombinant PLY and PLY-deficient *S. pneumoniae* [38]. Since IFN-γ produced by NK cells is important for the instruction of Th1 responses [23], our demonstration of NK cells producing IFN-γ in the lungs of infected mice could suggest subsequent promotion of pathogen-specific Th1 responses. Likewise, it has been suggested that IL-17A derived from γδ T cells can promote Th17 responses [24] and we demonstrate that there are IL-17A-producing γδ T cells in the lungs of mice infected with pneumococcus. Both of these effects were strongly dependent on pneumolysin, suggesting that the toxin is a key factor in pneumococcus-induced IFN-γ secretion by NK cells and IL-17A production by γδ T cells. Furthermore, since IL-23R signalling promotes the expansion and maintenance of γδ T cells in response to intracellular bacterial infection [39], the ability of pneumolysin to promote both IL-1 and IL-23 production is likely to contribute strongly to these γδ T cell responses.

During pneumococcal infection, PLY may synergize with pneumococcal or endogenous danger signals to induce the secretion of inflammatory cytokines. Reduced concentrations of plasma IL-6 have been reported from mice infected with PLY-deficient *S. pneumoniae* compared to isogenic wild-type pneumococci [40]. In addition, IL-1 and TNF-α are induced during murine pneumococcal disease [41] and elevated levels of both have been reported in patients with pneumococcal meningitis. Notably, studies using mice deficient in IL-1 receptor type 1 (IL-1R1^−/−^) [42] or IL-1β [27] have shown the importance of IL-1 in conferring resistance to pneumococcal meningitis and pneumonia respectively. The latter study found that IL-1β, but not IL-1α, plays a major role in resistance to pneumococcal infection. Therefore, the inflammasomes, which are required for IL-1β processing and secretion, are likely to be crucial components of the host defense to *S. pneumoniae*. Shoma *et al*. recently reported that recombinant PLY stimulated the secretion of IL-1α, IL-1β and IL-18 in a caspase-1 dependent manner in macrophages and that this was augmented by co-stimulation with pneumococci [38]. Importantly, we show here for the first time that the enhancement of IL-1β secretion by PLY is NLRP3-dependent. The ability of live pneumococci to promote IL-1β secretion by DC is also strongly dependent on NLRP3. One of the key observations in this study is that NLRP3 is required for protective immunity against pneumococcal infection. The first demonstration of a role for NLRP3 in defence against a Gram-negative pathogen, *Salmonella typhimurium*, was recently described [43], but our study is the first report to show that NLRP3 is required for protection against a Gram-positive bacterium.

NLRP3 activation by PLY required lysosomal damage and the release of cathepsin B, as inhibitors of these processes reduced PLY-induced IL-1β secretion. Phagosomal rupture has previously been shown to be important in NLRP3 inflammasome activation by other compounds including silica crystals, aluminium salts and microparticles [31], [32], [44] and a role for cathepsin B was recently reported in the promotion of IL-1β secretion by the pore-forming toxin tetanolysin O [45]. Furthermore, the ability of PLY to activate NLRP3 was dependent on the haemolytic activity of the toxin. Taken together, our data suggest that PLY-induced pore formation results in K^+^ efflux from DC and intracellular changes including lysosomal destabilization. The release of lysosomal products, such as cathepsin B, into the cytosol may promote the generation of danger signals, which are detected by NLRP3 or intermediary factors, resulting in inflammasome assembly and caspase-1 activation. Active caspase-1 could then process pro-IL-1β, which is generated in response to a second stimulus such as a TLR/NLR ligand, into mature IL-1β which is released from the cell.

Although a direct role for PLY in the induction of IL-1 or TNF-α during pneumococcal disease has not been established, the reduced inflammation and pathology in the lungs of mice infected with PLY-deficient pneumococci compared to wild type strains [4] suggests a diminished inflammatory cytokine response. In particular, lower levels of T cell infiltration and neutrophil recruitment into the lungs have been reported from mice infected with PLY-deficient *S. pneumoniae* compared to wild-type pneumococci [4]. TNF-α and IL-1β are two of the key cytokines involved in neutrophil recruitment in the lungs of mice infected intratracheally with wild-type *S. pneumoniae* [46]. Additionally, IL-17A plays a key role in neutrophil recruitment and its induction *in vivo* is strongly dependent on IL-1 [34], [36].

An additional major finding of this study is that the stimulatory effects of PLY on DC and splenocyte cytokine secretion are independent of TLR4. While we demonstrate that PLY can promote expression of costimulatory molecules and proinflammatory cytokines in DC, these effects are independent of TLR4. We also show that PLY exerts adjuvant effects, promoting antibody responses against a co-administered antigen in both wild-type and TLR4-defective mice, indicating that the immunostimulatory effects of PLY *in vivo* do not require TLR4. Therefore, further studies into the involvement of pathogen recognition receptors in sensing PLY in innate cells are warranted.

*Streptococcus pneumoniae* is a pathogen of significant clinical importance and understanding its interaction with the immune system is crucial. We propose that upon infection with *S. pneumoniae*, PLY, in synergy with pneumococcal PAMPs promotes the secretion of proinflammatory cytokines, particularly IL-1β, that promote an inflammatory response and mediate protective immunity. We identify PLY as a novel NLRP3 inflammasome activator and show that the ability of the live bacterium to promote IL-1β secretion is also strongly NLRP3-dependent. More importantly, NLRP3 is required for protective immunity against respiratory pneumococcal infection.

## Materials and Methods

### Ethics statement

Experiments on mice carried out at Trinity College Dublin were conducted under Irish Department of Health guidelines with ethical approval from the TCD ethics committee. Mice experiments carried out at the University of Leicester were done under UK Home Office guidelines with ethical approval from the UK Home Office.

### Reagents

CpG ODN 1826 was from Oligos Etc. *E. coli* LPS, Serotype R515 was obtained from Alexis Biochemicals while all other TLR agonists were obtained from InvivoGen.

Recombinant PLY was expressed in *E. coli* and purified as previously described [47]. Unless otherwise stated the specific haemolytic activity of PLY was 100,000 HU/mg. The toxin was passed three times through an EndoTrap endotoxin removal column (Profos AG, Germany) after which LPS was undetectable using the PyroGene Recombinant Factor C assay (Lonza; detection limit 0.01 EU/ml). *S. pneumoniae* serotype 2, strain D39 (NCTC 7466), was obtained from the National Collection of Type Culture, London, UK. The pneumolysin-negative mutant, PLN-A, was made by insertion duplication mutagenesis as described previously [2]. Heat-killed *S. pneumoniae* (HkSp) D39 was obtained by boiling 1×10^8^ CFU in phosphate buffered saline (PBS) for 20 minutes and checking for viability by colony counts and plate streaking. PLY W433F was a gift from The Netherlands Vaccine Institute.

### Mice for culture of macrophages, DC or splenocytes

Female BALB/c, C57BL/6, C3H/HeN and C3H/HeJ mice were obtained from Harlan Olac (UK) and were used at 9–16 weeks old. NLRP3^−/−^ mice were bred in the Bioresources Unit in Trinity College Dublin. Animals were maintained according to the regulations of the EU and the UK or the Irish Department of Health as appropriate. Caspase1^−/−^ DC were kindly provided by Dr. Katherine Fitzgerald (UMass, USA).

### Culture of DC and BMDM

DC and BMDM were prepared by culturing murine bone marrow cells using protocols adapted from Lutz *et al.* [48] and Davies *et al.* [49]. Briefly, bone marrow cells were flushed aseptically from the femurs and tibia of mice. For culture of DC, cells were grown in RPMI 1640 medium (Biosera) containing 8% v/v fetal calf serum (FCS; Biosera), 100 U/ml penicillin, 100 µg/ml streptomycin, and 100 mM L-glutamine (Gibco) and supplemented with supernatant from a granulocyte-macrophage colony-stimulating factor (GM-CSF)-expressing cell line (final concentration of 20 ng/ml GM-CSF). Macrophages were grown in Dulbecco's Modified Eagle's Medium containing 20 ng/ml M-CSF. Cultures were maintained in a humidified atmosphere (5% CO_2_) at 37°C, and medium was replaced on days 3, 6 and 8 for DC or days 2 and 4 for BMDM. On day 6 (BMDM) or day 10 (DC) of culture, cells were plated and stimulated with PLY and/or TLR agonists 24 hours later.

### Effect of PLY on DC maturation and cytokine production

DC (6.25×10^5^/ml) were cultured at 37°C for 24 h with medium, PLY or W433F alone, TLR and NLR agonists (PAM3Csk4, LPS, zymosan, MDP, CpG) alone, HkSp alone or PLY or W433F and TLR agonists or HkSp together. In certain experiments, DC were incubated with YVAD-fmk (Bachem), KCl (Sigma), CA-074-ME (Sigma) or Bafilomycin A (Sigma) 30 mins before the addition of PLY and/or TLR agonists. At the end of the incubation, supernatants were removed and cytokine concentrations were determined by ELISA using pairs of antibodies purchased from BD Biosciences (IL-6 and IL-12p40) or R&D Systems (IL-1α, IL-1β, TNF-α, IL-12p70, IL-10 or IL-23) according to the manufacturer's specifications. Alternatively, cells were recovered and used for immunofluorescence analysis or Western Blotting. The expression of DC surface markers was assessed using fluorochrome-labelled antibodies against murine CD80, CD86, I-A/I-E, CD11c and CD40 (BD Biosciences). After blocking for 10 minutes on ice with anti-mouse CD16/CD32 (BD Biosciences) followed by incubation with antibodies for 30 minutes on ice, cells were washed and immunofluorescence analysis was performed on a CyAN (Dako) flow cytometer using FlowJo software (Tree Star). For Western blot analysis, cells were lysed in Laemmli sample buffer, samples were boiled and proteins separated by SDS/PAGE. Caspase-1 p10 was detected as previously described [44].

### Effect of *S. pneumoniae* on DC cytokine production

DC (6.25×10^5^/ml) from C57BL/6 and NLRP3^−/−^ mice were cultured at 37°C in RPMI 1640 medium (Biosera) containing 8% v/v FCS (Biosera) and 100 mM L-glutamine (Gibco) in the absence of antibiotics. Cells were stimulated with PBS or with wild-type (D39; 10 bacteria:1 DC) or PLY-deficient *S. pneumoniae* (PLN; 10 bacteria:1 DC) in PBS. Following 24 h incubation, supernatants were removed and analysed for cytokine production by ELISA.

### Adoptive transfer of PLY and antigen-stimulated DC

DC (1×10^6^/ml) were incubated overnight with medium alone or with KLH (10 µg/ml) with or without PLY (1 µg/ml). After extensive washing, cells (1×10^5^/mouse) were injected subcutaneously into the footpads of mice. After 7 days, splenocytes were isolated and resuspended at 2×10^6^ cells/ml in RPMI medium, supplemented with 8% v/v FCS. Cells were cultured in triplicate with KLH (2 or 50 µg/ml) or with medium only as a negative control. Proliferative responses were assessed after 4 days of culture by [^3^H]-thymidine incorporation.

### Effect of PLY on cytokine production by spleen cells

Splenocytes were isolated from C3H/HeN or C3H/HeJ mice by passing spleens through a 70 µm cell strainer (BD Falcon). After lysing erythrocytes in 0.88% w/v NH_4_Cl solution for 5 minutes and washing, cells were incubated in 96-well plates (1×10^6^/ml) in RPMI medium with 8% v/v FCS plus antibiotics. Splenocytes were stimulated for 72 hours with PLY in the presence of plate-bound anti-CD3 (BD Biosciences) or HkSp and in some cases for a further 24 hours with PMA (Sigma Aldrich) and ionomycin (Sigma Aldrich). Supernatants were removed after 72 or 96 hours and tested by ELISA for IL-10, IL-17A (R&D Systems), IFN-γ and IL-5 (BD Biosciences).

### Intranasal infection of mice with wild-type and PLY-deficient pneumococci

The pneumococcal infection studies were done at the University of Leicester under UK Home Office guidelines or in Trinity College Dublin under Irish Department of Health guidelines. Wild-type *S. pneumoniae* serotype 2 strain D39, NCTC 7466 (NCTC, London, UK) was used. An isogenic PLY-negative mutant, PLN-A, has been described previously [2]. Pneumococci were cultured on blood agar base with 5% v/v horse blood, or in brain heart infusion broth (BHI; Oxoid, Basingstoke, UK) with 20% v/v foetal bovine serum (Gibco, Paisley, UK), supplemented with 1 µg/ml erythromycin (Sigma, Poole, UK) for PLN-A. Before use, pneumococci were passaged through mice, as described previously [4] and aliquots stored at −80°C. When required, the suspension was thawed at room temperature, and bacteria were harvested by centrifugation before resuspension in sterile PBS.

Female outbred MF1, BALB/c, C57BL/6 (Harlan, Bichester, UK) or NLRP3^−/−^ (bred in the Bioresources Unit of Trinity College Dublin) mice (9–10 weeks old, 30–35 g) were used for infection studies. Mice were lightly anaesthetized, as described previously [4], and 50 µl PBS containing 1×10^6 ^CFU *S. pneumoniae* was administered into the nostrils. The inoculum dose was confirmed by viable count following infection. At pre-chosen time intervals following infection, mice were sacrificed and lungs removed, weighed, and homogenised with an Ultra-Turrax T8 homogeniser (IKA, Germany). CFU bacterial counts were determined by viable count on blood agar plates as described previously [4]. IFN-γ and IL-17A concentrations in lung homogenates were measured by ELISA (BD Biosciences).

### Analysis of intracellular IL-17A and IFN-γ production in the lungs of infected mice

Forty-eight hours post-infection of BALB/c mice with D39 wild-type *S. pneumoniae* (as described in the previous section), mice were sacrificed, lungs removed and cells isolated as described previously [4]. For intracellular cytokine staining, lung cells were cultured for five hours at 5×10^5^ cells/well in round-bottomed 96-well plates in RPMI 10% FCS supplemented with GolgiPlug (BD Biosciences), according to manufacturer's instructions, to block cellular secretion of cytokines. Media was further supplemented with 500 ng/ml ionomycin and 50 ng/ml PMA. Following culture, cells were washed and stained with antibodies against extracellular markers (anti-CD3, anti-CD4, anti-CD8, anti-NKp46, anti-NK1.1, anti-CD45 or anti-γδTCR; BioLegend or eBioscience). Cells were then incubated with Fix/Perm solution (BD Biosciences) for 20 minutes before washing in Perm/Wash buffer (BD Biosciences). Staining with antibodies against intracellular cytokines was performed with anti-IL17A and anti-IFN-γ antibodies diluted in Perm/Wash buffer. After staining, cells were washed and resuspended in PBS 3% (v/v) FCS prior to data collection.

### Statistical analyses

Unless otherwise stated, data were compared by one-way ANOVA. The Tukey-Kramer multiple-comparison test was used to identify significant differences between individual groups.

### Methods for supporting data

Methods for supporting data are described in Methods S1.

## Supporting Information

Methods S1 Supplementary Materials and Methods
(0.02 MB RTF)

Figure S1 Sensitivity of splenocytes to toxic concentrations of PLY is independent of TLR4. Splenocytes (1×10^6^ cells/ml) from either C3H/HeN or C3H/HeJ mice were incubated with medium alone or with various concentrations of PLY (1.6 ng/ml-5 µg/ml) for 6, 24 or 72 hours. After stimulation, cells were washed, stained with Annexin V-FITC and propidium iodide (PI; 1 µg/ml) and analysed by flow cytometry for dye uptake. Cell death is expressed as the percentage of cells that took up PI out of the total cell number and is representative of data from two independent experiments. (A) Representative dot plots showing AnnexinV and/or PI positive splenocytes from C3H/HeJ mice following stimulation with PLY for 6 hours. (B) Cell death in splenocytes from both C3H/HeN and C3H/HeJ mice stimulated with PLY for 24 hours. (C) Cell death in splenocytes from both C3H/HeN and C3H/HeJ mice stimulated with PLY for 72 hours.
(0.80 MB TIF)

Figure S2 Growth of wild-type and pneumolysin-deficient *S. pneumoniae* in the lungs of infected mice. (A) Acute pneumonia model. MF1 mice were infected as described in Fig. 2A. Bacterial CFU were determined in the lungs of infected mice at 0, 24 and 48 hours post-infection. ** P<0.01 vs. WT. (B) Resolving pneumonia model. BALB/c mice were infected intranasally as described in Fig. 2B. Bacterial CFU were determined in the lungs of infected mice at 0, 24 and 48 hours post-infection. *, P<0.05, ** P<0.01 vs. WT.
(0.14 MB TIF)

Figure S3 Sensitivity of DC to toxic concentrations of PLY is independent of TLR4. DC (6.25×10^5^ cells/ml) from either C3H/HeN or C3H/HeJ mice were incubated with medium alone or with various concentrations of PLY (0.1–6 µg/ml) for 6 or 24 hours. After stimulation, cells were washed, stained with Annexin V-FITC and propidium iodide (PI; 1 µg/ml) and analysed by flow cytometry for dye uptake. Cell death is expressed as the percentage of cells that took up PI out of the total cell number and is representative of data from three independent experiments. (A) Cell death in DC from C3H/HeJ mice. (B) Cell death in DC from both C3H/HeN and C3H/HeJ mice stimulated with 6 µg/ml PLY. (C) Representative dot plots showing AnnexinV and/or PI positive DC from C3H/HeJ mice following stimulation with PLY for 6 hours.
(0.51 MB TIF)

Figure S4 PLY enhances antibody titres to co-administered KLH independently of TLR4. (A) and (B) Female BALB/c mice were immunized s.c. in the footpad with PBS, KLH (10 µg), or KLH (10 µg) and PLY (10 µg). (A) Splenocytes and popliteal lymph node cells, isolated 7 days later, were stimulated with KLH (10 or 50 µg/ml), medium alone or PMA and anti-CD3. Supernatants were removed after 3 days and were tested for IL-5, IFN-γ, and IL-17 by immunoassay. Results represent the mean (+ SEM) of three mice per group and are representative of at least three independent experiments. (B) Anti-KLH IgG, IgG1, IgG2a and IgG2b titres were determined in serum, recovered 7 days post-immunization, by ELISA. * P<0.05, ** P<0.01 and *** P<0.001, Student's t test. (C) C3H/HeN and C3H/HeJ mice were immunized s.c. in the footpad with PBS, KLH (10 µg), or KLH (10 µg) and PLY (1 µg). Anti-KLH IgG1 and IgG2a titres were determined in serum, recovered 7 days post-immunization, by ELISA. * P<0.05 and ** P<0.01, Student's t test.
(0.29 MB TIF)

Figure S5 PLY synergizes with heat-killed pneumococci to enhance IL-6 and TNF-α production by BMDM. BMDM (6.25×10^5^/ml) from C57BL/6 mice were incubated with PLY (1 µg/ml) for 1 hour before the addition of HkSp (10 bacteria:1 cell). IL-6 and TNF-α concentrations were measured by ELISA in supernatants removed after 24 hours. Values are represented as mean cytokine concentrations (+ SEM) from triplicate samples. ** P<0.01 and *** P<0.001 vs. bacteria alone.
(0.11 MB TIF)

Figure S6 PLY promotes IL-1β secretion in DC in a NLRP6- and NLRP12-independent manner. DC from wild-type C57BL/6, NLRP6^−/−^ or NLRP12^−/−^ mice were incubated with PLY (0.5 µg/ml) for 1 hour before the addition of LPS (500 pg/ml). IL-1β concentrations were quantified in supernatants after 24 hours and are presented as mean values (+ SEM) from triplicate cultures. *** P<0.001 vs. LPS alone.
(0.19 MB TIF)

Figure S7 The production of pro-IL-1β in response to TLR activation is not compromised in NLRP3^−/−^ DC. DC from wild-type C57BL/6 (represented as c57) or NLRP3^−/−^ (represented as ko) mice were stimulated with PLY (0.5 µg/ml) for 1 hour before the addition of PAM3Csk (10 µg/ml) and incubated for 24 hours. Pro-IL-1β in cell lysates was detected by Western blot.
(0.49 MB TIF)

Figure S8 The ability of PLY to enhance IL-1β secretion is compromised in ASC^−/−^ DC compared to wild-type DC. DC from wild-type C57BL/6 or ASC^−/−^ mice were incubated with PLY (1 µg/ml) for 1 hour before the addition of Pam3CSK (10 µg/ml). After 24 hours supernatants were assayed for IL-1β, IL-1α and TNF-α. * P<0.05 and *** P<0.001 vs. PAM3 alone.
(0.23 MB TIF)

## References

[1] Tweten RK (2005). Cholesterol-dependent cytolysins, a family of versatile pore-forming toxins.. Infect Immun.

[2] Berry AM, Yother J, Briles DE, Hansman D, Paton JC (1989). Reduced virulence of a defined pneumolysin-negative mutant of Streptococcus pneumoniae.. Infect Immun.

[3] Canvin JR, Marvin AP, Sivakumaran M, Paton JC, Boulnois GJ (1995). The role of pneumolysin and autolysin in the pathology of pneumonia and septicemia in mice infected with a type 2 pneumococcus.. J Infect Dis.

[4] Kadioglu A, Gingles NA, Grattan K, Kerr A, Mitchell TJ (2000). Host cellular immune response to pneumococcal lung infection in mice.. Infect Immun.

[5] Kadioglu A, Taylor S, Iannelli F, Pozzi G, Mitchell TJ (2002). Upper and lower respiratory tract infection by Streptococcus pneumoniae is affected by pneumolysin deficiency and differences in capsule type.. Infect Immun.

[6] Feldman C, Munro NC, Jeffery PK, Mitchell TJ, Andrew PW (1991). Pneumolysin induces the salient histologic features of pneumococcal infection in the rat lung in vivo.. Am J Respir Cell Mol Biol.

[7] Mitchell TJ, Andrew PW, Saunders FK, Smith AN, Boulnois GJ (1991). Complement activation and antibody binding by pneumolysin via a region of the toxin homologous to a human acute-phase protein.. Mol Microbiol.

[8] Cockeran R, Steel HC, Mitchell TJ, Feldman C, Anderson R (2001). Pneumolysin potentiates production of prostaglandin E(2) and leukotriene B(4) by human neutrophils.. Infect Immun.

[9] Cockeran R, Theron AJ, Steel HC, Matlola NM, Mitchell TJ (2001). Proinflammatory interactions of pneumolysin with human neutrophils.. J Infect Dis.

[10] Kadioglu A, Coward W, Colston MJ, Hewitt CR, Andrew PW (2004). CD4-T-lymphocyte interactions with pneumolysin and pneumococci suggest a crucial protective role in the host response to pneumococcal infection.. Infect Immun.

[11] Braun JS, Hoffmann O, Schickhaus M, Freyer D, Dagand E (2007). Pneumolysin causes neuronal cell death through mitochondrial damage.. Infect Immun.

[12] Houldsworth S, Andrew PW, Mitchell TJ (1994). Pneumolysin stimulates production of tumor necrosis factor alpha and interleukin-1 beta by human mononuclear phagocytes.. Infect Immun.

[13] Malley R, Henneke P, Morse SC, Cieslewicz MJ, Lipsitch M (2003). Recognition of pneumolysin by Toll-like receptor 4 confers resistance to pneumococcal infection.. Proc Natl Acad Sci U S A.

[14] Srivastava A, Henneke P, Visintin A, Morse SC, Martin V (2005). The apoptotic response to pneumolysin is Toll-like receptor 4 dependent and protects against pneumococcal disease.. Infect Immun.

[15] Ratner AJ, Hippe KR, Aguilar JL, Bender MH, Nelson AL (2006). Epithelial cells are sensitive detectors of bacterial pore-forming toxins.. J Biol Chem.

[16] Koga T, Lim JH, Jono H, Ha UH, Xu H (2008). Tumor suppressor cylindromatosis acts as a negative regulator for Streptococcus pneumoniae-induced NFAT signaling.. J Biol Chem.

[17] Benton KA, Paton JC, Briles DE (1997). The hemolytic and complement-activating properties of pneumolysin do not contribute individually to virulence in a pneumococcal bacteremia model.. Microb Pathog.

[18] Branger J, Knapp S, Weijer S, Leemans JC, Pater JM (2004). Role of Toll-like receptor 4 in gram-positive and gram-negative pneumonia in mice.. Infect Immun.

[19] Rubins JB, Pomeroy C (1997). Role of gamma interferon in the pathogenesis of bacteremic pneumococcal pneumonia.. Infect Immun.

[20] Nakamatsu M, Yamamoto N, Hatta M, Nakasone C, Kinjo T (2007). Role of interferon-gamma in Valpha14+ natural killer T cell-mediated host defense against Streptococcus pneumoniae infection in murine lungs.. Microbes Infect.

[21] Lu YJ, Gross J, Bogaert D, Finn A, Bagrade L (2008). Interleukin-17A mediates acquired immunity to pneumococcal colonization.. PLoS Pathog.

[22] Steinman RM, Hemmi H (2006). Dendritic cells: translating innate to adaptive immunity.. Curr Top Microbiol Immunol.

[23] Martin-Fontecha A, Thomsen LL, Brett S, Gerard C, Lipp M (2004). Induced recruitment of NK cells to lymph nodes provides IFN-gamma for T(H)1 priming.. Nat Immunol.

[24] Sutton CE, Lalor SJ, Sweeney CM, Brereton CF, Lavelle EC (2009). Interleukin-1 and IL-23 induce innate IL-17 production from gammadelta T cells, amplifying Th17 responses and autoimmunity.. Immunity.

[25] Gingles NA, Alexander JE, Kadioglu A, Andrew PW, Kerr A (2001). Role of genetic resistance in invasive pneumococcal infection: identification and study of susceptibility and resistance in inbred mouse strains.. Infect Immun.

[26] Kadioglu A, Andrew PW (2005). Susceptibility and resistance to pneumococcal disease in mice.. Brief Funct Genomic Proteomic.

[27] Kafka D, Ling E, Feldman G, Benharroch D, Voronov E (2008). Contribution of IL-1 to resistance to Streptococcus pneumoniae infection.. Int Immunol.

[28] Martinon F, Mayor A, Tschopp J (2009). The inflammasomes: guardians of the body.. Annu Rev Immunol.

[29] Walev I, Reske K, Palmer M, Valeva A, Bhakdi S (1995). Potassium-inhibited processing of IL-1 beta in human monocytes.. EMBO J.

[30] Gurcel L, Abrami L, Girardin S, Tschopp J, van der Goot FG (2006). Caspase-1 activation of lipid metabolic pathways in response to bacterial pore-forming toxins promotes cell survival.. Cell.

[31] Hornung V, Bauernfeind F, Halle A, Samstad EO, Kono H (2008). Silica crystals and aluminum salts activate the NALP3 inflammasome through phagosomal destabilization.. Nat Immunol.

[32] Yoshimori T, Yamamoto A, Moriyama Y, Futai M, Tashiro Y (1991). Bafilomycin A1, a specific inhibitor of vacuolar-type H(+)-ATPase, inhibits acidification and protein degradation in lysosomes of cultured cells.. J Biol Chem.

[33] Martin B, Hirota K, Cua DJ, Stockinger B, Veldhoen M (2009). Interleukin-17-producing gammadelta T cells selectively expand in response to pathogen products and environmental signals.. Immunity.

[34] Iwakura Y, Nakae S, Saijo S, Ishigame H (2008). The roles of IL-17A in inflammatory immune responses and host defense against pathogens.. Immunol Rev.

[35] Stockinger B, Veldhoen M (2007). Differentiation and function of Th17 T cells.. Curr Opin Immunol.

[36] Sutton C, Brereton C, Keogh B, Mills KH, Lavelle EC (2006). A crucial role for interleukin (IL)-1 in the induction of IL-17-producing T cells that mediate autoimmune encephalomyelitis.. J Exp Med.

[37] Zhu J, Paul WE (2008). CD4 T cells: fates, functions, and faults.. Blood.

[38] Shoma S, Tsuchiya K, Kawamura I, Nomura T, Hara H (2008). Critical involvement of pneumolysin in production of interleukin-1alpha and caspase-1-dependent cytokines in infection with Streptococcus pneumoniae in vitro: a novel function of pneumolysin in caspase-1 activation.. Infect Immun.

[39] Riol-Blanco L, Lazarevic V, Awasthi A, Mitsdoerffer M, Wilson BS (2010). IL-23 receptor regulates unconventional IL-17-producing T cells that control bacterial infections.. J Immunol.

[40] Benton KA, Everson MP, Briles DE (1995). A pneumolysin-negative mutant of Streptococcus pneumoniae causes chronic bacteremia rather than acute sepsis in mice.. Infect Immun.

[41] Bergeron Y, Ouellet N, Deslauriers AM, Simard M, Olivier M (1998). Cytokine kinetics and other host factors in response to pneumococcal pulmonary infection in mice.. Infect Immun.

[42] Zwijnenburg PJ, van der Poll T, Florquin S, Roord JJ, Van Furth AM (2003). IL-1 receptor type 1 gene-deficient mice demonstrate an impaired host defense against pneumococcal meningitis.. J Immunol.

[43] Broz P, Newton K, Lamkanfi M, Mariathasan S, Dixit VM (2010). Redundant roles for inflammasome receptors NLRP3 and NLRC4 in host defense against Salmonella.. J Exp Med.

[44] Sharp FA, Ruane D, Claass B, Creagh E, Harris J (2009). Uptake of particulate vaccine adjuvants by dendritic cells activates the NALP3 inflammasome.. Proc Natl Acad Sci U S A.

[45] Chu J, Thomas LM, Watkins SC, Franchi L, Nunez G (2009). Cholesterol-dependent cytolysins induce rapid release of mature IL-1{beta} from murine macrophages in a NLRP3 inflammasome and cathepsin B-dependent manner.. J Leukoc Biol.

[46] Jones MR, Simms BT, Lupa MM, Kogan MS, Mizgerd JP (2005). Lung NF-kappaB activation and neutrophil recruitment require IL-1 and TNF receptor signaling during pneumococcal pneumonia.. J Immunol.

[47] Gilbert RJ, Rossjohn J, Parker MW, Tweten RK, Morgan PJ (1998). Self-interaction of pneumolysin, the pore-forming protein toxin of Streptococcus pneumoniae.. J Mol Biol.

[48] Lutz MB, Kukutsch N, Ogilvie AL, Rossner S, Koch F (1999). An advanced culture method for generating large quantities of highly pure dendritic cells from mouse bone marrow.. J Immunol Methods.

[49] Davies JQ, Gordon S (2005). Isolation and culture of murine macrophages.. Methods Mol Biol.

