# Supplementary material for: Pneumolysin Activates the NLRP3 Inflammasome and Promotes Proinflammatory Cytokines Independently of TLR4
Source: PLoS Pathog. 2010 Nov 11;6(11):e1001191. doi: 10.1371/journal.ppat.1001191 (PMC2978728; doi:10.1371/journal.ppat.1001191)
Supplement: Methods S1 — Supplementary Materials and Methods (0.02 MB RTF) [file ppat.1001191.s001.rtf]

Additional Materials and Methods
ASC-/-, NLRP6-/- and NLRP12-/- DC were kindly provided by Dr. Kate Fitzgerald, UMass, Worcester, USA.
Assessment of the adjuvanticity of PLY. BALB/c, C3H/HeN or C3H/HeJ mice were immunized s.c. in the footpad with PBS as control or with either KLH (10 µg) alone or with PLY (1 or 10 µg).  After 7 days, spleens and popliteal LN were removed to test for KLH-specific cytokine production, and/or serum was recovered to assess KLH-specific antibody responses.  Spleen cells (2×10^6^ cells/ml) or popliteal lymph node cells (1×10^6^ cells/ml) from immunized or naïve mice were cultured in triplicate wells of 96-well microtitre plates with KLH (10-50 ìg/ml) or with phorbal myristate acetate (PMA; Sigma 20 ng/ml) and anti-mouse CD3 (PharMingen; 1 ìg/ml) or medium only as positive and negative controls, respectively.  Supernatants from the culture plates were removed after 72 h and the concentrations of IFN-ã, IL-5 and IL-17 were determined by specific immunoassays using commercially available pairs of antibodies (BD Biosciences).  KLH-specific serum antibodies were quantified by ELISA using plate-bound KLH (5 µg/ml). Bound antibodies were detected using biotin-conjugated anti-mouse IgG (Sigma Aldrich), IgG1, IgG2a or IgG2b (BD Biosciences) followed by peroxidase-conjugated streptavidin (Sigma Aldrich). Antibody responses are expressed as the reciprocal of the serum dilution (log10) that gave an OD of 2 standard deviations above that obtained with naïve mouse serum.

Effect of PLY on splenocyte or DC death as determined by AnnexinV/PI staining. DC and splenocytes from C3H/HeN and C3H/HeJ mice stimulated with various concentrations of PLY for 6 hours, 24 hours and 72 hours were analysed for early signs of apoptosis and cell death by AnnexinV/PI staining. Briefly, cells were washed once in PBS and resuspended in PBS supplemented with CaCl2 (1.8 mM) and containing AnnexinV-FITC (BD Biosciences) according to manufacturer's recommendations. After 15 minutes incubation at room temperature, cells were washed and resuspended in PBS/CaCl2. Propidium iodide (PI; Sigma 1 ìg/ml) was added immediately prior to analysis on a CyAN (Dako) flow cytometer.

Detection of pro-IL-1â by Western blot. Pro-IL-1â was detected in cell lysates by Western blot using rat anti-mouse IL-1â (R&D Systems; 1 ìg/ml) followed by peroxidase-conjugated anti-rat IgG (Thermo Scientific).  
   

 
