# Supplementary figures and images for: Pneumolysin Activates the NLRP3 Inflammasome and Promotes Proinflammatory Cytokines Independently of TLR4
Source: PLoS Pathog. 2010 Nov 11;6(11):e1001191. doi: 10.1371/journal.ppat.1001191 (PMC2978728; doi:10.1371/journal.ppat.1001191)

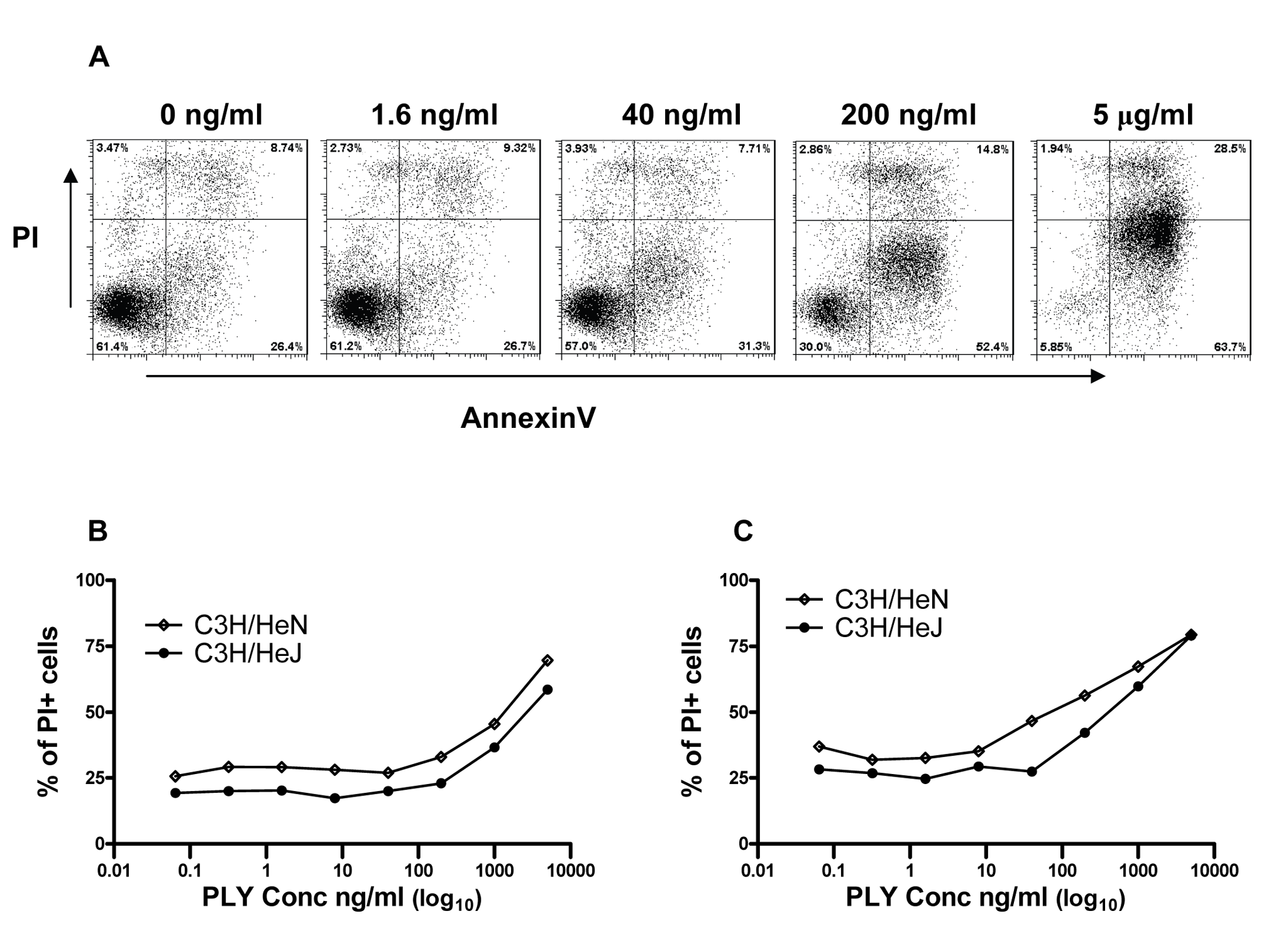

Supplement: Figure S1 — Sensitivity of splenocytes to toxic concentrations of PLY is independent of TLR4. Splenocytes (1×10^6^ cells/ml) from either C3H/HeN or C3H/HeJ mice were incubated with medium alone or with various concentrations of PLY (1.6 ng/ml-5 µg/ml) for 6, 24 or 72 hours. After stimulation, cells were washed, stained with Annexin V-FITC and propidium iodide (PI; 1 µg/ml) and analysed by flow cytometry for dye uptake. Cell death is expressed as the percentage of cells that took up PI out of the total cell number and is representative of data from two independent experiments. (A) Representative dot plots showing AnnexinV and/or PI positive splenocytes from C3H/HeJ mice following stimulation with PLY for 6 hours. (B) Cell death in splenocytes from both C3H/HeN and C3H/HeJ mice stimulated with PLY for 24 hours. (C) Cell death in splenocytes from both C3H/HeN and C3H/HeJ mice stimulated with PLY for 72 hours. (0.80 MB TIF) [file ppat.1001191.s002.tif]

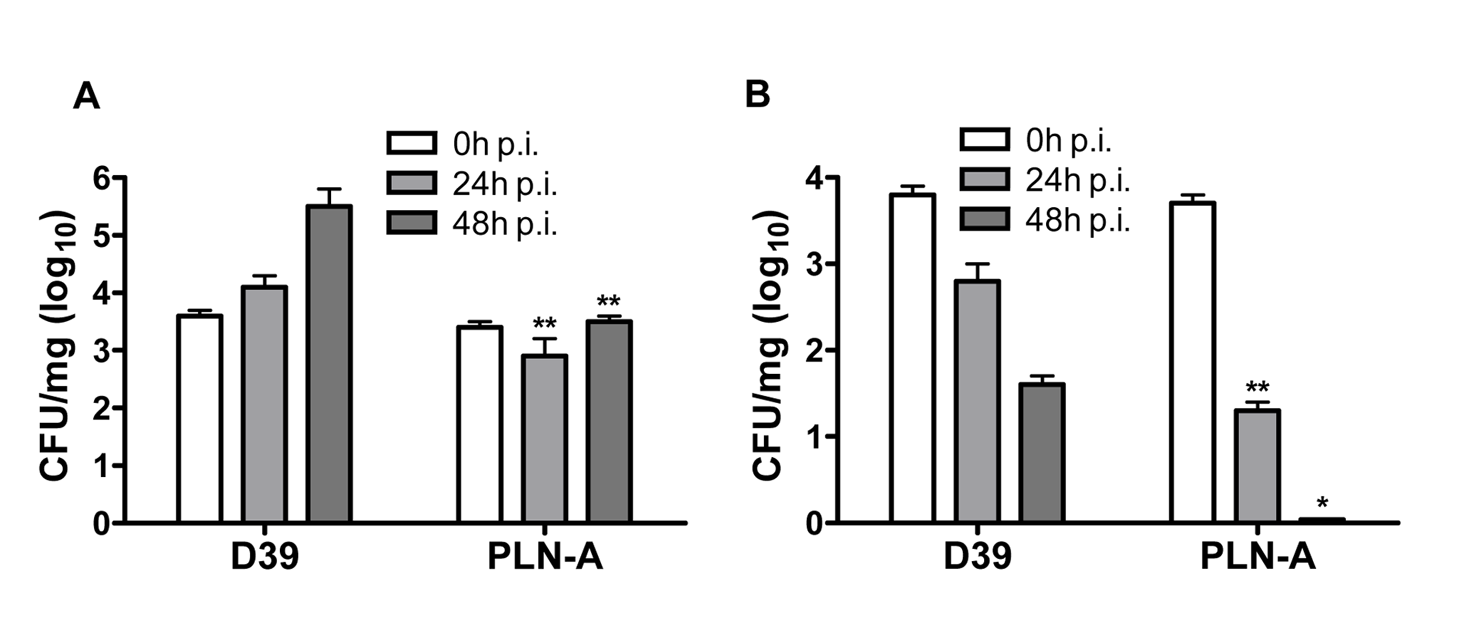

Supplement: Figure S2 — Growth of wild-type and pneumolysin-deficient S. pneumoniae in the lungs of infected mice. (A) Acute pneumonia model. MF1 mice were infected as described in Fig. 2A. Bacterial CFU were determined in the lungs of infected mice at 0, 24 and 48 hours post-infection. ** P<0.01 vs. WT. (B) Resolving pneumonia model. BALB/c mice were infected intranasally as described in Fig. 2B. Bacterial CFU were determined in the lungs of infected mice at 0, 24 and 48 hours post-infection. *, P<0.05, ** P<0.01 vs. WT. (0.14 MB TIF) [file ppat.1001191.s003.tif]

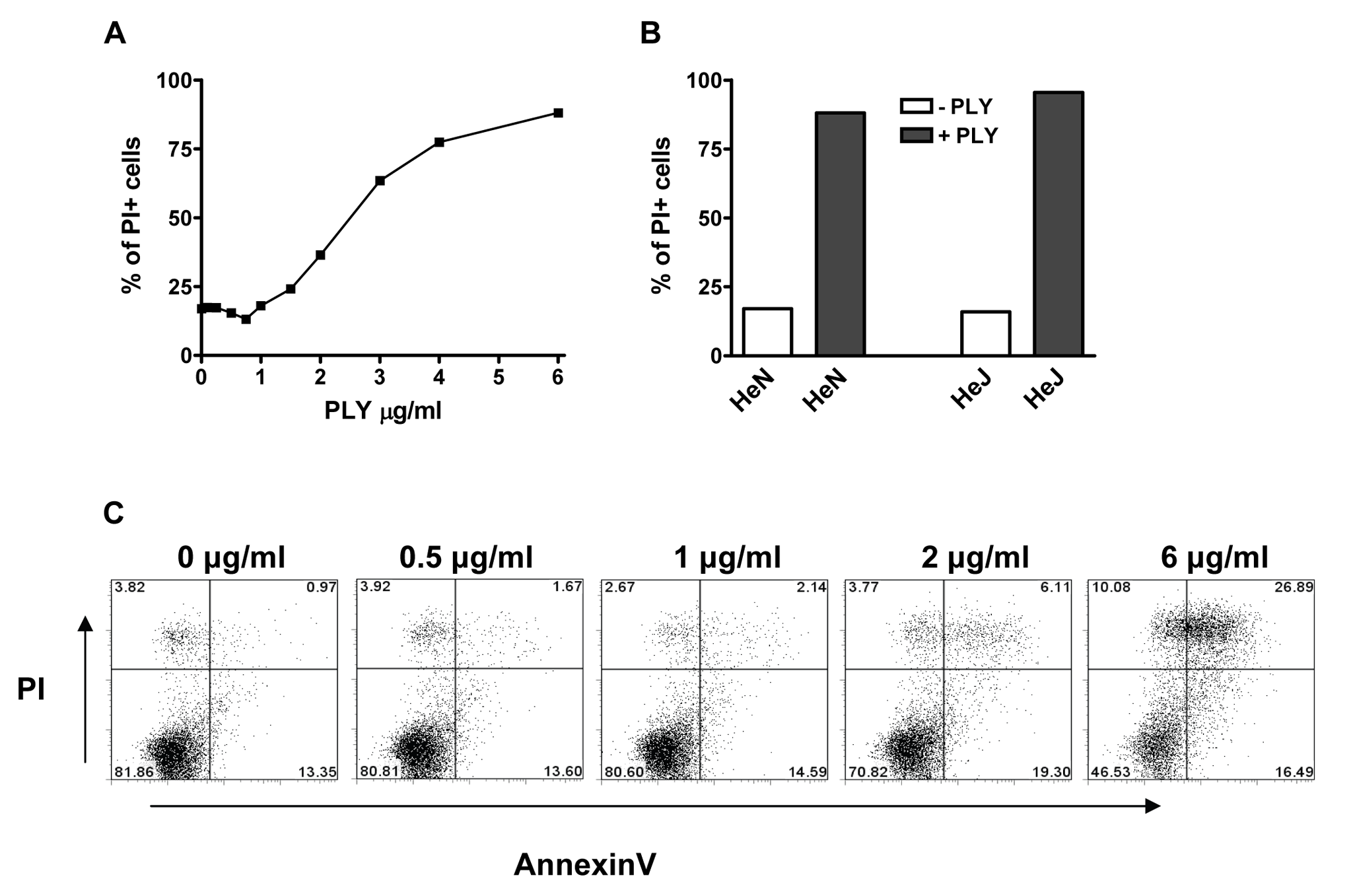

Supplement: Figure S3 — Sensitivity of DC to toxic concentrations of PLY is independent of TLR4. DC (6.25×10^5^ cells/ml) from either C3H/HeN or C3H/HeJ mice were incubated with medium alone or with various concentrations of PLY (0.1–6 µg/ml) for 6 or 24 hours. After stimulation, cells were washed, stained with Annexin V-FITC and propidium iodide (PI; 1 µg/ml) and analysed by flow cytometry for dye uptake. Cell death is expressed as the percentage of cells that took up PI out of the total cell number and is representative of data from three independent experiments. (A) Cell death in DC from C3H/HeJ mice. (B) Cell death in DC from both C3H/HeN and C3H/HeJ mice stimulated with 6 µg/ml PLY. (C) Representative dot plots showing AnnexinV and/or PI positive DC from C3H/HeJ mice following stimulation with PLY for 6 hours. (0.51 MB TIF) [file ppat.1001191.s004.tif]

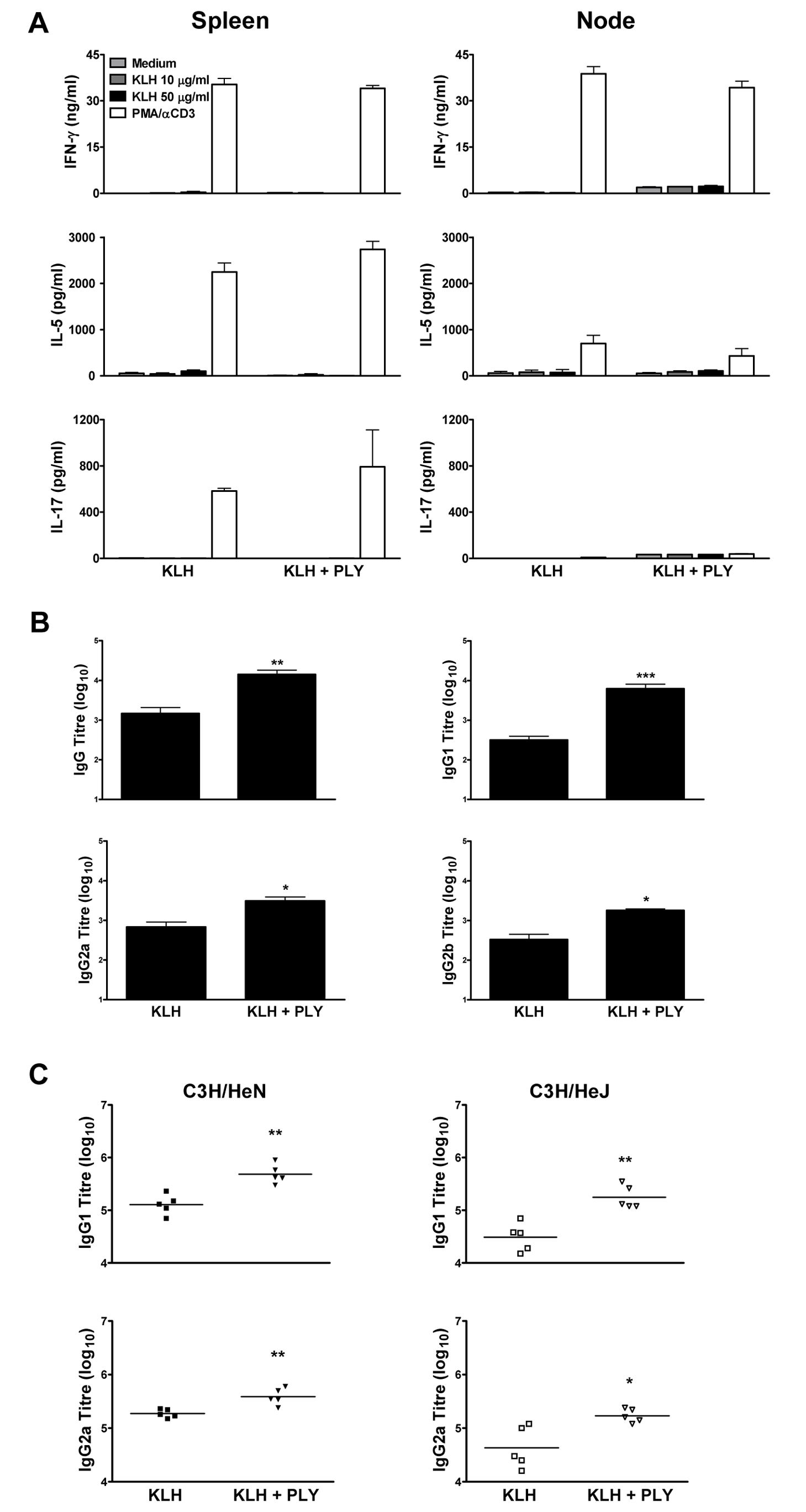

Supplement: Figure S4 — PLY enhances antibody titres to co-administered KLH independently of TLR4. (A) and (B) Female BALB/c mice were immunized s.c. in the footpad with PBS, KLH (10 µg), or KLH (10 µg) and PLY (10 µg). (A) Splenocytes and popliteal lymph node cells, isolated 7 days later, were stimulated with KLH (10 or 50 µg/ml), medium alone or PMA and anti-CD3. Supernatants were removed after 3 days and were tested for IL-5, IFN-γ, and IL-17 by immunoassay. Results represent the mean (+ SEM) of three mice per group and are representative of at least three independent experiments. (B) Anti-KLH IgG, IgG1, IgG2a and IgG2b titres were determined in serum, recovered 7 days post-immunization, by ELISA. * P<0.05, ** P<0.01 and *** P<0.001, Student's t test. (C) C3H/HeN and C3H/HeJ mice were immunized s.c. in the footpad with PBS, KLH (10 µg), or KLH (10 µg) and PLY (1 µg). Anti-KLH IgG1 and IgG2a titres were determined in serum, recovered 7 days post-immunization, by ELISA. * P<0.05 and ** P<0.01, Student's t test. (0.29 MB TIF) [file ppat.1001191.s005.tif]

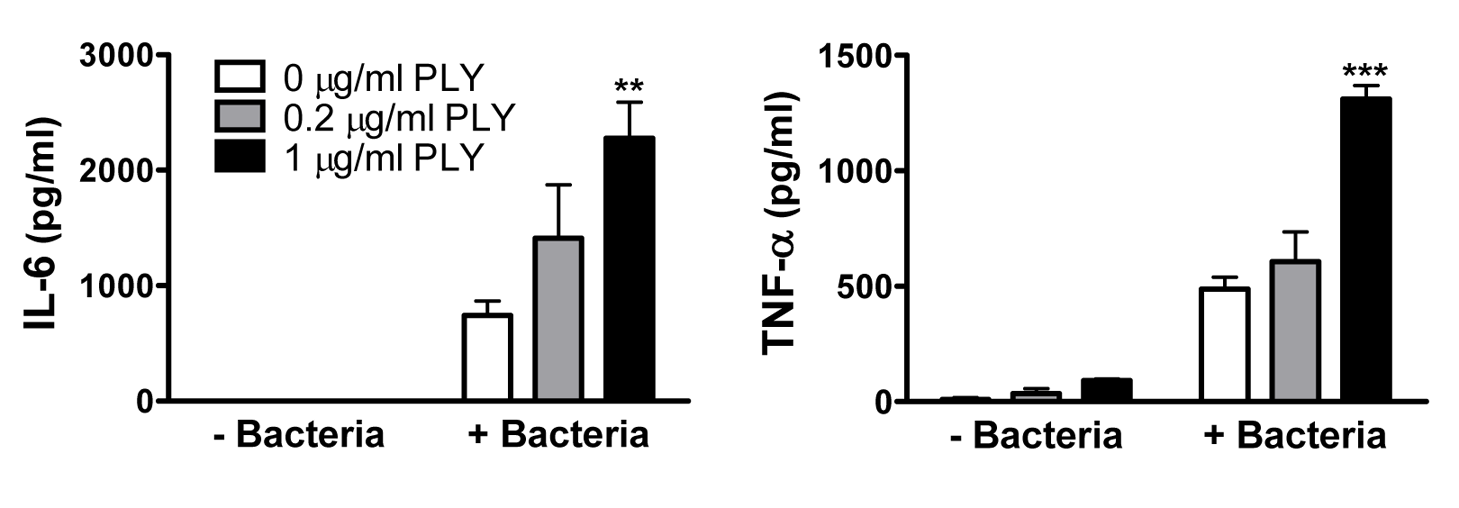

Supplement: Figure S5 — PLY synergizes with heat-killed pneumococci to enhance IL-6 and TNF-α production by BMDM. BMDM (6.25×10^5^/ml) from C57BL/6 mice were incubated with PLY (1 µg/ml) for 1 hour before the addition of HkSp (10 bacteria:1 cell). IL-6 and TNF-α concentrations were measured by ELISA in supernatants removed after 24 hours. Values are represented as mean cytokine concentrations (+ SEM) from triplicate samples. ** P<0.01 and *** P<0.001 vs. bacteria alone. (0.11 MB TIF) [file ppat.1001191.s006.tif]

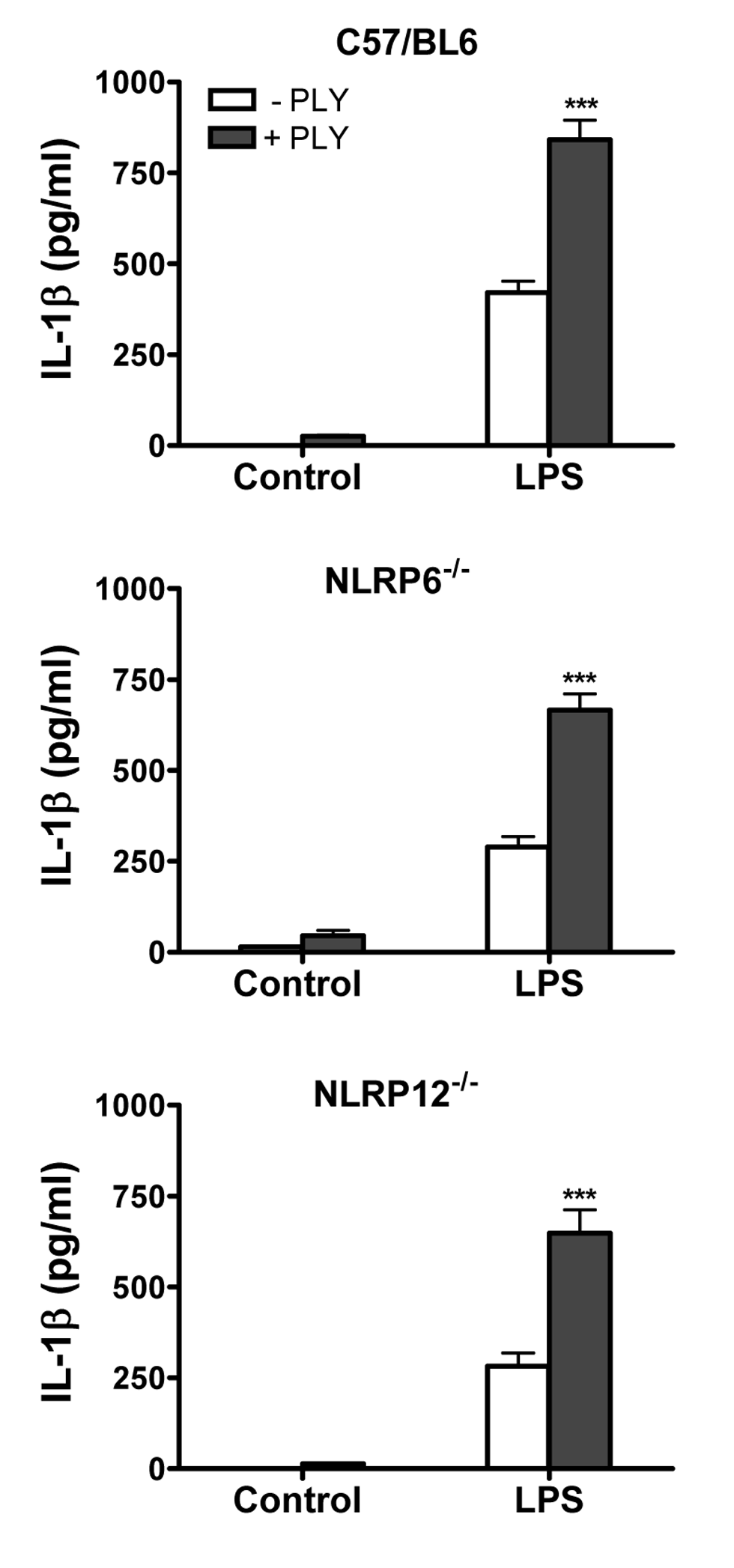

Supplement: Figure S6 — PLY promotes IL-1β secretion in DC in a NLRP6- and NLRP12-independent manner. DC from wild-type C57BL/6, NLRP6−/− or NLRP12−/− mice were incubated with PLY (0.5 µg/ml) for 1 hour before the addition of LPS (500 pg/ml). IL-1β concentrations were quantified in supernatants after 24 hours and are presented as mean values (+ SEM) from triplicate cultures. *** P<0.001 vs. LPS alone. (0.19 MB TIF) [file ppat.1001191.s007.tif]

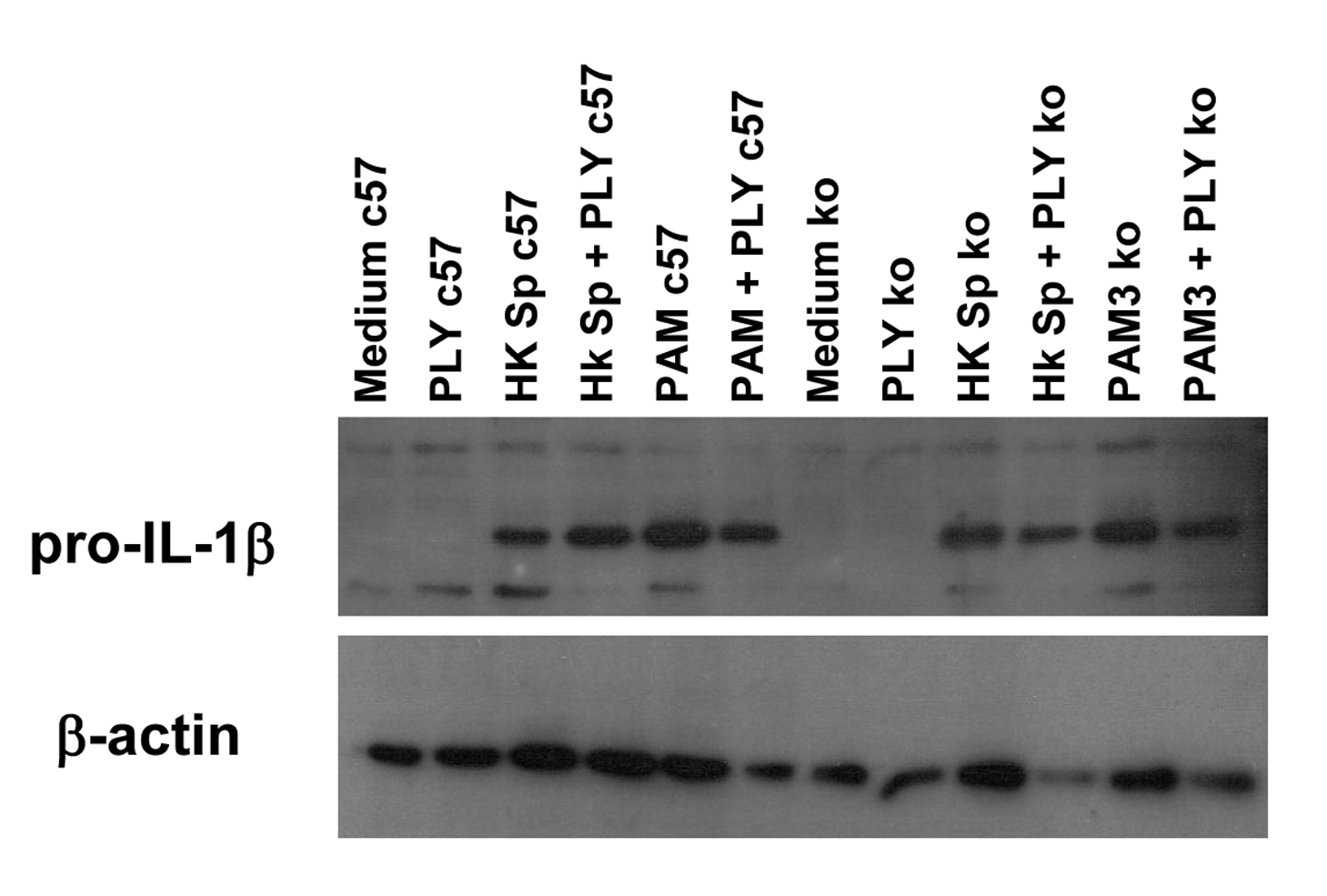

Supplement: Figure S7 — The production of pro-IL-1β in response to TLR activation is not compromised in NLRP3−/− DC. DC from wild-type C57BL/6 (represented as c57) or NLRP3−/− (represented as ko) mice were stimulated with PLY (0.5 µg/ml) for 1 hour before the addition of PAM3Csk (10 µg/ml) and incubated for 24 hours. Pro-IL-1β in cell lysates was detected by Western blot. (0.49 MB TIF) [file ppat.1001191.s008.tif]

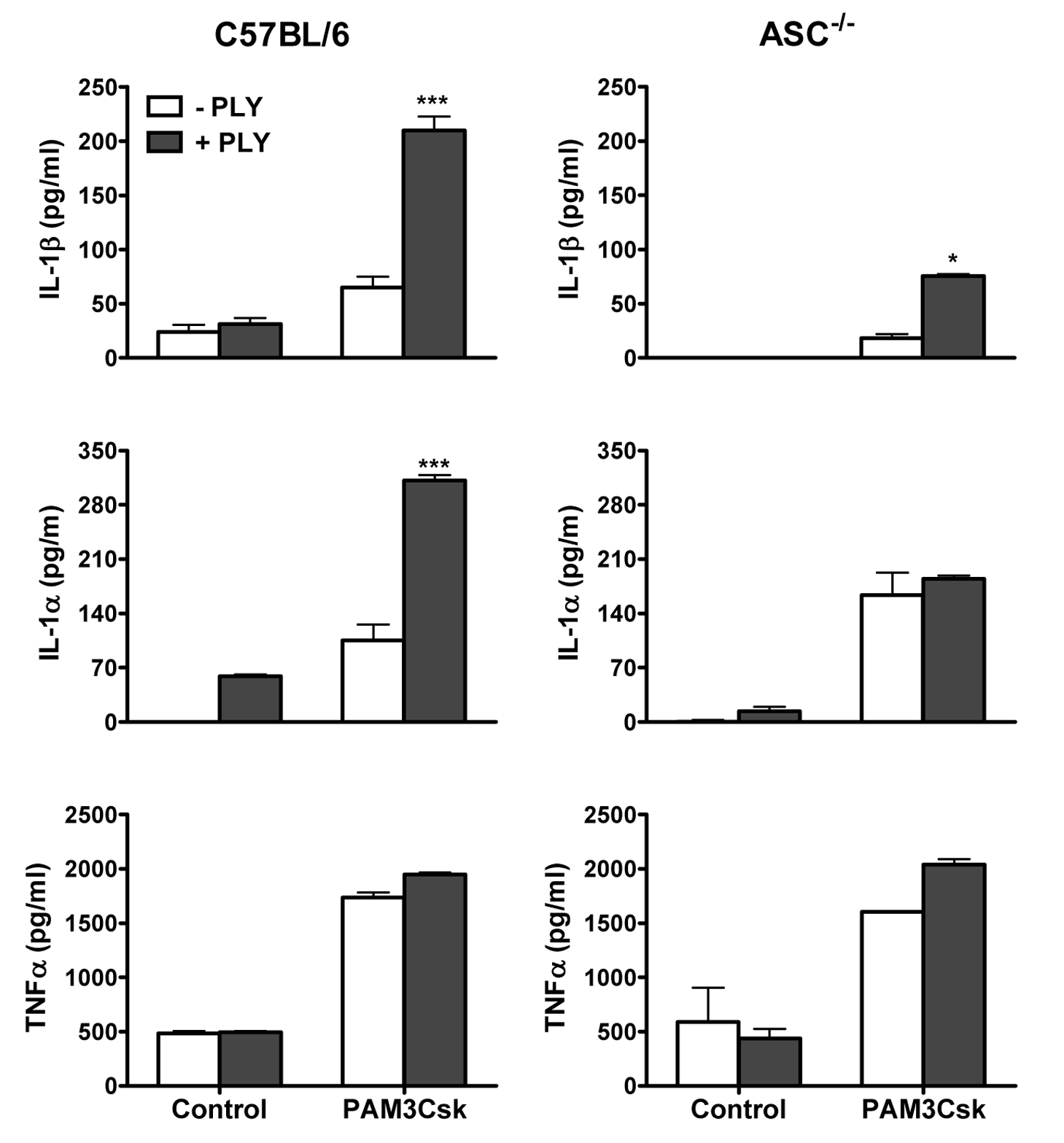

Supplement: Figure S8 — The ability of PLY to enhance IL-1β secretion is compromised in ASC−/− DC compared to wild-type DC. DC from wild-type C57BL/6 or ASC−/− mice were incubated with PLY (1 µg/ml) for 1 hour before the addition of Pam3CSK (10 µg/ml). After 24 hours supernatants were assayed for IL-1β, IL-1α and TNF-α. * P<0.05 and *** P<0.001 vs. PAM3 alone. (0.23 MB TIF) [file ppat.1001191.s009.tif]
